# Dietary choline, *via* gut microbe- generated trimethylamine-N- oxide, aggravates chronic kidney disease-induced cardiac dysfunction by inhibiting hypoxia-induced factor 1α

**DOI:** 10.3389/fphys.2022.996166

**Published:** 2022-11-03

**Authors:** Feifei Xie, Xin Zhen, Zhuoliang Liu, Xiaomei Chen, Zhuanhua Liu, Miaomiao Zhou, Zhanmei Zhou, Zheng Hu, Fengxin Zhu, Qiaobing Huang, Lei Zhang, Jing Nie

**Affiliations:** ^1^ The State Key Laboratory of Organ Failure Research, National Clinical Research Center of Kidney Disease, Key Laboratory of Organ Failure Research (Ministry of Education), Division of Nephrology, Nanfang Hospital, Southern Medical University, Guangzhou, China; ^2^ Nephrology Division, The Third Affiliated Hospital of Sun Yat-sen University, Guangzhou, Guangdong, China; ^3^ Department of Pathophysiology, Guangdong Provincial Key Laboratory of Shock and Microcirculation, School of Basic Medical Sciences, Southern Medical University, Guangzhou, China

**Keywords:** trimethylamine-N-oxide, chronic kidney disease, cardiac dysfunction, angiogenesis, hypoxia-induced factor 1α, dietary choline

## Abstract

Chronic kidney disease (CKD) is a global public health problem that shortens lifespan primarily by increasing the risk of cardiovascular diseases. Trimethylamine-N-oxide (TMAO), a gut microbiota-derived toxin produced by metabolizing high-choline or carnitine foods, is associated with cardiovascular events in patients with CKD. Although the deleterious effect of TMAO on CKD-induced cardiac injury has been confirmed by various researches, the mechanisms remain unclear. Here, we tested the hypothesis that TMAO aggravates CKD-induced cardiac injury and explores the potential mechanism. CD1 mice underwent 5/6 nephrectomy to induce CKD, and then fed with a diet supplemented with choline (1.2% total) for 8 weeks. Serum TMAO levels were elevated in CKD mice compared with SHAM group, and higher TMAO levels were found in choline-supplemented CKD mice compared with CKD group. Dietary choline aggravated CKD-induced cardiac dysfunction, and reducing TMAO levels *via* medicinal charcoal tablets improved cardiac dysfunction. RNA-seq analysis revealed that dietary choline affected cardiac angiogenesis in CKD mice. Reduced cardiac capillary density and expressions of angiogenesis-related genes were observed in choline-treated CKD mice. Furthermore, dietary choline inhibited cardiac Hif-1α protein level in CKD mice, and Hif-1α stabilizer FG-4592 could improve cardiac angiogenesis and dysfunction in CKD mice on a high-choline diet. In conclusion, these data indicate that dietary choline, *via* gut microbe-generated TMAO, inhibits cardiac angiogenesis by reducing Hif-1α protein level, ultimately aggravates cardiac dysfunction in CKD mice.

## Introduction

Chronic kidney disease (CKD) is a global public health problem with a high burden of morbidity and mortality, and cardiovascular disease (CVD) is the major cause of mortality in patients with CKD (London, 2003; Tonelli et al., 2006; GBD Chronic Kidney Disease Collaboration, 2020). Uremic cardiomyopathy (UC), the major phenotype of fatal cardiac disease in patients with end stage renal disease, characterized by left ventricular hypertrophy, cardiac fibrosis, capillary rarefaction, and both systolic and diastolic dysfunction (Wang et al., 2017; Wang and Shapiro, 2019), is associated with sudden cardiac death and recurrent heart failure in CKD patients (Zoccali, 2010; Green et al., 2011; Collins et al., 2015). Therefore, there is an urgent need to understand the mechanisms and identify effective therapeutic targets of UC for improving the prognosis of CKD patients.

Numerous clinical and experimental studies document that the pathogenesis of UC is complex (Hung et al., 2015; Hung et al., 2017; Wang et al., 2017; Wang and Shapiro, 2019; Bi et al., 2020). Among them, insufficient angiogenesis plays an important role (Semple et al., 2011; Wang and Shapiro, 2019). Angiogenesis is an essential event involved in ischemic heart disease, which promotes the growth of new capillary blood vessels and restores the blood flow of ischemic tissue (Tabibiazar and Rockson, 2001), and is regulated by secreted angiogenic growth factors such as vascular endothelial growth factor-A (VEGFA) and angiopoietin-1 (Angpt1), *etc.* (Breier et al., 1997). It has been shown that capillary growth failed to keep pace with cardiomyocyte hypertrophy, resulting in decreased density in experimental and clinical studies of CKD (Amann et al., 1992; Amann et al., 1998). However, the pathogenesis of inadequate capillary adaptation in UC is unclear.

A growing body of evidence has linked the cardiovascular risk of CKD to an accumulation of uremic toxins (UTs) that occurs with progression of CKD (Liabeuf et al., 2010; Stubbs et al., 2016; Hung et al., 2017). Microbiota toxins, such as indoxyl sulfate (IS) and p-cresyl sulfate (PCS), are well-known to contribute to several cardiovascular pathologies, including accelerated atherosclerosis (Zhang et al., 2018; Nakano et al., 2019) and hyperthrombotic state (Yang et al., 2017). Elevated indolic solutes levels are thought to involved in suppression of endothelial cell survival and migration, which are critical for angiogenesis process (Dou et al., 2004; Hung et al., 2016). In recent years, trimethylamine-N-oxide (TMAO), another gut-derived uremic toxin, has been extensively studied. It is produced by gut flora *via* metabolizing food containing choline, lecithin, betaine, and carnitine (Subramaniam and Fletcher, 2018). TMAO is predominantly excreted by the kidney, so it is significantly elevated in patients with CKD (Tang et al., 2015a). In the past several years, an association of elevated levels of systemic TMAO with various human diseases, including cardiovascular diseases (Tang et al., 2013; Tang et al., 2015b; Troseid et al., 2015; Suzuki et al., 2017; Li et al., 2018; Nie et al., 2018; Hsu et al., 2022) and kidney diseases (Fang et al., 2021; Lai et al., 2022) has been reported. Many studies have revealed that TMAO promotes vascular inflammation, induces atherosclerosis, as well as enhances platelet hyperreactivity and thrombosis risk (Hartiala et al., 2014; Seldin et al., 2016; Zhu et al., 2016). It has been demonstrated that decreasing plasma TMAO levels by targeting choline TMA lyase, can attenuate atherosclerosis and thrombosis (Wang et al., 2015; Roberts et al., 2018). Although some clinical studies have shown that elevated TMAO levels is a risk factor of cardiovascular events (Tang et al., 2013; Stubbs et al., 2016; Shafi et al., 2017; Fu et al., 2021), it is largely unclear whether TMAO directly promotes the progression of CKD-induced cardiac injury.

In the present study, we investigated the role of TMAO on CKD-induced cardiac dysfunction and the underlying mechanism. In addition, we explored whether targeting TMAO or its related mechanisms can improve CKD-induced cardiac dysfunction.

## Materials and methods

### Animals

According to the previous literature (Kennedy et al., 2008; Leelahavanichkul et al., 2010), we selected CD1 mice as the experimental animals. Male CD1 mice were purchased from the Vital River Laboratories, Beijing, China. All mice were housed in a specific pathogen-free condition with 12/12 h light/dark cycle and free access to food and water. All animal experiments were reviewed and approved by the Ethics Committee for Animal Experiments of the Southern Medical University.

### Protocol

To evaluate the role of TMAO in CKD-induced cardiac injury, we fed CD1 mice with supplementary 1.2% choline in diet for 8 weeks to raise their serum TMAO levels according to previous literature (Organ et al., 2016; Shuai et al., 2020). CD1 mice were randomly divided into three groups as follows (*n* = 6 per group): SHAM group, CKD group and CKD + Choline group. CKD mice were subjected to five-sixths nephrectomy (5/6 Nx) as described previously (Gschwend et al., 2002). After the operation, SHAM and CKD mice were given a standard chow diet (TP 3001M, Trophic Animal Feed High-Tech Co. Ltd., Jiangsu, China), and CKD + Choline mice were fed with the same diet supplemented with choline (1.2% total). At 8 weeks after treatment, cardiac function was evaluated by echocardiograms, then all mice were sacrificed and serum, heart, and kidney tissues were collected for various analyses.

In addition, in order to observe the role of medicinal charcoal tablets (MCT) on cardiac injury in CKD mice treated with dietary choline, we performed another animal experiment by treating mice with MCT for 8 weeks (*n* = 6 per group). Mice were randomly divided into two groups as follows: CKD + Choline group and CKD + Choline + C group. The CKD + Choline group was treated as before, and CKD + Choline + C group were fed with the same diet supplemented with choline (1.2% total) and 4% MCT (Changtian Pharmaceutical Co., Ltd., Hebei, China).

Afterwards, to assess the role of FG-4592 on cardiac injury in CKD mice treated with dietary choline, mice were randomly divided into four groups as follows: SHAM group, CKD group, CKD + Choline group and CKD + Choline + FG-4592 group. The first three groups were treated as before, and CKD + Choline + FG-4592 group were administered to dietary choline (1.2% total) and intraperitoneal injection of FG-4592 (Selleck Chemicals, S1007). The FG-4592 was dissolved in DMSO at the concentration of 50 mg/ml and further diluted in PBS to 1 mg/ml. The mice were treated by intraperitoneal injection three times per week with 10 mg/kg/day FG-4592 for 3 weeks in CKD + Choline + FG-4592 group at 5 weeks after the operation. At 8 weeks after 5/6 Nx, cardiac function was evaluated by echocardiograms, then mice were sacrificed and serum, and heart tissues were collected for various analyses.

### Echocardiography

Cardiac function was assessed by Doppler echocardiography (VisualSonics Vevo2100 Imaging system, Toronto, Ontario, Canada) with a 21-MHz transducer (MS400) before mice were sacrificed. Mice were mildly anesthetized by inhaling 3.0% isoflurane and oxygen at rate of 1 L/min. Images were standardized to the short axis view at the LV mid-papillary level.

### Assessment of renal function

Serum creatinine (Scr) and blood urea nitrogen (BUN) concentration were measured by an automated chemistry analyzer (AU480; Beckman Coulter, Brea, CA, United States).

### Quantification of trimethylamine-N-oxide levels

Serum concentrations of TMAO were quantified by stable isotope dilution liquid chromatography tandem mass spectrometry (6460 Series Triple Quadrupole LC/MS; Agilent, CA, United States) as described previously (Wang et al., 2014).

### Histology and immunohistochemistry staining

Paraffin-embedded kidney sections (4 μm) were subjected to hematoxylin and eosin (H&E) and Masson trichrome staining according to standard protocols. Tubular injury was graded with H&E-stained sections ranging from 0 to 4 according to the degree of tubular necrosis, dilatation, or cell swelling: 0, less than 5%; 1, 5–25%; 2, 25–50%; 3, 50–75%; and 4, over 75% (Lai et al., 2022). At least 10 randomly chosen fields in the cortex region under the microscope (×200) were evaluated for each animal in a blinded manner, and an average score was calculated.

Interstitial fibrosis was assessed using Image-Pro Plus 6.0 (Media Cybernetics, Silver Spring, MD, United States) on Masson trichrome stained sections. Ten visual fields (×400) were randomly selected for each animal and evaluated by a background subtraction method. Quantification is presented as the ratio of optical density of positive staining compared to the entire spectrum.

Immunohistochemistry staining was performed on 6 μm heart sections. After antigen retrieval, sections were incubated with the primary antibodies against CD31 (Cell Signaling Technology, Beverly, MA, United States). Images were taken by an Olympus BX51 microscope (Olympus, Tokyo, Japan). Endothelial cells were considered as capillaries, as previous described (Lu et al., 2017). We counted CD31-positive areas about ten random fields (×400) per section, and then calculated the mean number of micro vessels per field (capillary density).

## Real-time PCR

Total RNA from heart tissues was extracted using TRIzol reagent according to the manufacturer’s instructions (Invitrogen). Superscript III First-Strand Synthesis SuperMix (Invitrogen) was used for reverse transcription of 1 μg of total RNA. PCR was performed using SYBR Green Master Mix (Applied Biosystems) and the Applied Biosystems 7500 fast Real-time PCR system. The expression levels of mRNAs were calculated after normalizing with GAPDH by the comparative CT method (2^−ΔΔCt^). The primer sequences used in the experiments were described as follows:

Mouse ANP: forward, 5′-ACC​TGC​TAG​ACC​ACC​TGG​AG-3′; reverse, 5′-CCTTGGCTGTTATCT TCGGTACCGG-3′; Mouse BNP: forward, 5′-GAG​GTC​ACT​CCT​ATC​CTC​TGG-3′; reverse, 5′- GCC​ATT​TCC​TCC​GAC​TTT​TCT​C -3′; Mouse β-MHC: forward, 5′- TGG​ATT​CTC​AAA​CGT​GTC​TAG​TGA -3′; reverse, 5′-GCATTCTCCTGCTGT TTCCTT-3′; Mouse α-MHC: forward,5′- GCCCAGTACCTCCGA AAGTC -3′; reverse, 5′- ATCAGGCACGAAGC ACTCC -3′; Mouse SERCA2: forward, 5′- AGA​TGG​TCC​TGG​CAG​ATG​AC -3′; reverse, 5′- GTC​CAG​GTC​TGG​AGG​ATT​GA -3′;

Mouse VEGFA: forward, 5′- GGA​GTC​TGT​GCT​CTG​GGA​TT -3′; reverse, 5′- AGA​ACC​AAC​CTC​CTC​AAA​CCG -3′; Mouse VEGFR2: forward, 5′- TTTGGCAAATACAAC CCTTCAGA -3′; reverse, 5′- GCT​CCA​GTA​TCA​TTT​CCA​ACC​A -3′; Mouse Angpt1: forward, 5′- ATC​CCG​ACT​TGA​AAT​ACA​ACT​GC -3′; reverse, 5′- CTG​GAT​GAT​GAA​TGT​CTG​ACG​AG -3′;

Mouse Tie2: forward, 5′- CTA​AAT​TTG​ACT​TGG​CAA​CCG​A -3′; reverse, 5′- TCT​GCT​GAT​CAC​TTG​TTG​TTT​G -3′; Mouse Slit2: forward, 5′- GGC​AGA​CAC​TGT​CCC​TAT​CG -3′; reverse, 5′- ATC​TAT​CTT​CGT​GAT​CCT​CGT​GA -3′; Mouse Robo1: forward, 5′- AAC​GGG​AGA​GTG​AAG​TCG​C -3′; reverse, 5′- TCTTTCCTCCATCGA ACTGTAGG -3′; Mouse GAPDH: forward, 5′- TGA​CCT​CAA​CTA​CAT​GGT​CTA​CA -3′; reverse, 5′- CTT​CCC​ATT​CTC​GGC​CTT​G -3′.

### Western blot analysis

Heart tissues were lysed in lysis buffer for 30 min on ice. Western blot analysis was performed following procedure as described previously. The following primary antibodies were used: anti-SERCA2 (Abcam, Cambridge, United Kingdom), anti-CD31 (Cell Signaling Technology, Beverly, MA, United States), anti-Hif-1α (Proteintech, Rosemont, United States) and anti-GAPDH (Proteintech, Rosemont, United States).

### RNA sequencing and bioinformatic analysis

Transcriptome analysis was performed by Gene *Denovo* Biotechnology Co. (Guangzhou, China). Briefly, total RNAs of the heart tissue in CKD or CKD + Choline mice were extracted using the RNAiso Plus Reagent (TaKaRa, Mountain View, CA, United States). Then, mRNA was enriched by Oligo (dT) beads fragmented into short fragments and reverse-transcribed with random primers. The cDNA fragments were purified, end repaired, poly (A) added, and ligated to sequencing adapters. The ligation products were size-selected, PCR-amplified, and sequenced. Raw reads were filtered, mapped to the reference genome, reconstructed to transcripts, and annotated. The gene expression level was quantified and differently expressed genes were analyzed using edgeR (version 3.12.1) (http://www.r-project.org/). Principal component analysis (PCA) was performed with R package gmodels in this experience. Genes with a fold change> 0 and *p* value < 0.05 were considered significant differentially expressed genes. Finally, we used the toppgene online analysis tool (https://toppgene.cchmc.org/) to perform enrichment analysis of differential genes in multiple databases, including GO, pathway and other databases.

### Statistical analysis

Data were expressed as mean ± SD. Results were analyzed for statistical variance using independent Student’s t-tests or one-way ANOVA analysis where appropriate. A two-sided *p* value < 0.05 was considered to be statistically significant (SPSS software, version 18.0; SPSS, Inc., IL).

## Results

### Dietary choline (1.2%) exacerbates cardiac dilatation and dysfunction after 5/6 Nx in CD1 mice

**FIGURE 1:**
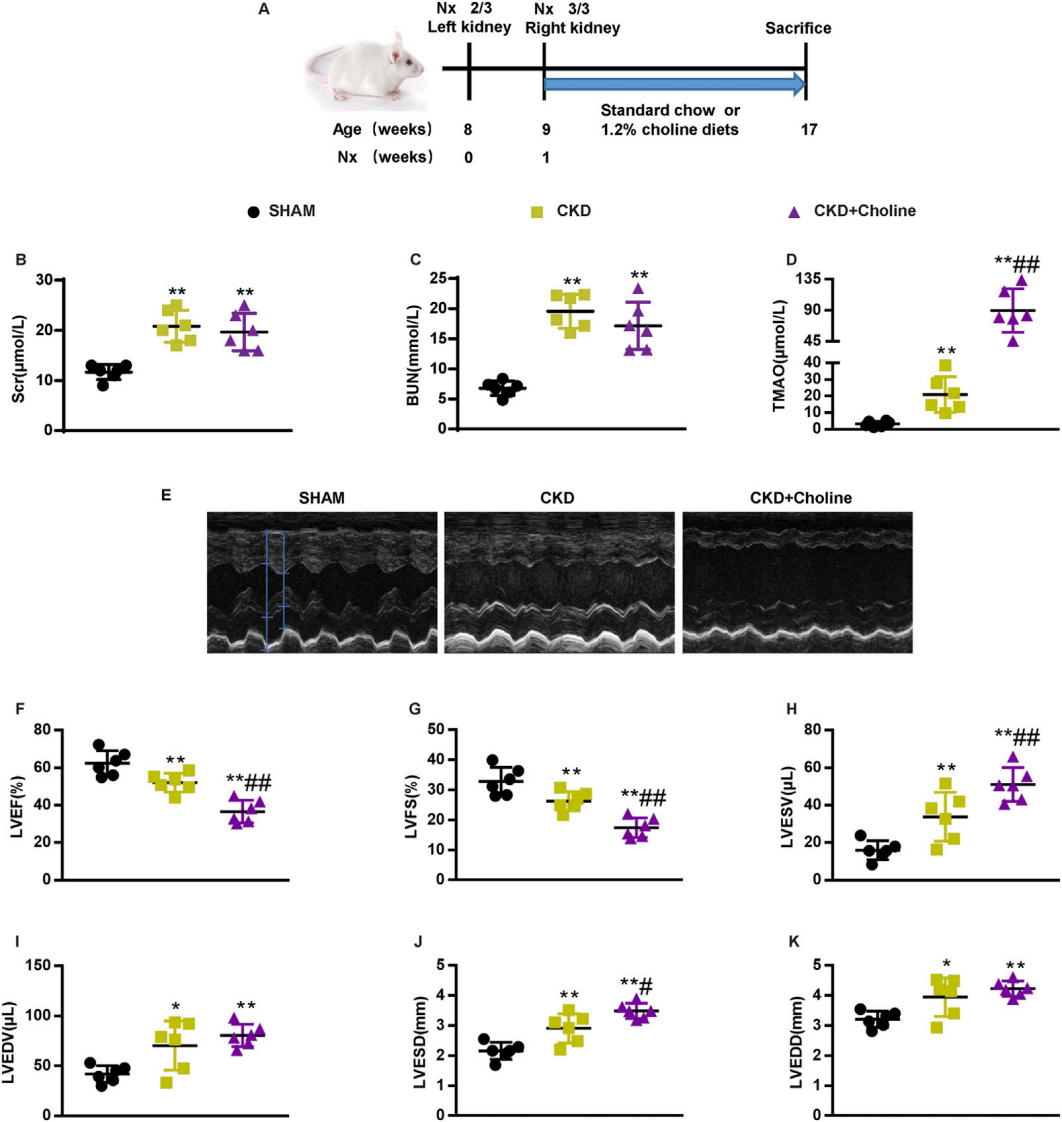
Dietary choline (1.2%) exacerbates cardiac dilation and dysfunction after 5/6 nephrectomy (5/6 Nx) in CD1 mice when compared with a control diet. **(A)** Schematic design of CKD animal model (5/6 Nx) and treatments **(B)** The serum level of TMAO. **(C)** The serum level of serum creatinine (Scr). **(D)** The serum level of blood urea nitrogen (BUN). **(E)** Representative M-mode echocardiograms for each group. **(F–K)** Echocardiographic quantification of left ventricular ejection fraction (LVEF %), LV fractional shortening (LVFS %), LV end-systolic volume (LVESV; in μL), LV end-diastolic volume (LVEDV; in μL), LV end-systolic diameter (LVESD; in mm), and LV end-diastolic diameter (LVEDD; in mm). The data are presented as the mean ± SD. ^*^ *p* < 0.05 and ^**^ *p* < 0.01 *vs.* SHAM group; ^#^ *p* < 0.05 and ^##^ *p* < 0.01 *vs.* CKD group. *n* = 6 in each group.

To investigate the role of TMAO in CKD-induced cardiac injury, we generated CKD mice by conducting 5/6 Nx in male CD1 mice. After the operation, 5/6 Nx mice were then randomly divided into two groups and given either a standard chow diet (0.1% choline) or the same diet supplemented with 1.2% choline for 8 weeks (Figure 1A). As shown in Figures 1B,C, compared with SHAM mice, serum creatinine (Scr) and blood urea nitrogen (BUN) were both significantly elevated in the CKD group, while dietary choline did not increase the levels of Scr and BUN. HE and Masson trichrome staining showed renal tubular necrosis, protein casts, inflammatory cell infiltration as well as renal interstitial fibrosis in the CKD mice. However, these histopathological changes were not further aggravated by dietary choline (Supplementary Figure S1A–C). Next, serum levels of TMAO were measured. As expected, serum levels of TMAO were significantly increased in CKD mice compared with those of the SHAM group, while those of CKD + Choline group were prominently higher than those of the CKD group (Figure 1D). Taken together, these data indicate that 1.2% dietary choline can further increase the levels of TMAO in the CKD mice, but could not accelerate CKD progression.

Then, cardiac structure and function in mice were assessed by echocardiography at 8 weeks post-surgery. Representative echocardiograms were shown in Figure 1E. Compared with SHAM mice, CKD mice displayed left ventricular (LV) dilatation, as evidenced by significantly increased LV end-systolic diameter (LVESD) and LV end-diastolic diameter (LVEDD). CKD mice also showed LV dysfunction, as confirmed by significantly decreased LV ejection fraction (LVEF) and LV fractional shortening (LVFS), and increased LV end-systolic volume (LVESV) and LV end-diastolic volume (LVEDV). Mice that received 1.2% dietary choline exhibited markedly worse cardiac function in almost all parameters measured when compared with the CKD mice. An accelerated progression of cardiac dilatation, as proved by remarkably increased LVESD, were observed in the CKD + Choline group; LVEDD were trending toward an increase in the CKD + Choline group *versus* CKD group. In addition, we also observed worse LV dysfunction, as revealed by significantly decreased LVEF and LVFS, and increased LVESV in the CKD + Choline group (Figures 1F–K). Collectively, these data clearly demonstrate a detrimental effect of supplemental dietary choline on LV structure and function in CKD model.

### Dietary choline (1.2%) promotes changes in markers of cardiac hypertrophy and inhibits cardiac sarcoplasmic reticulum Ca2^+^-ATPase 2 expression

**FIGURE 2:**
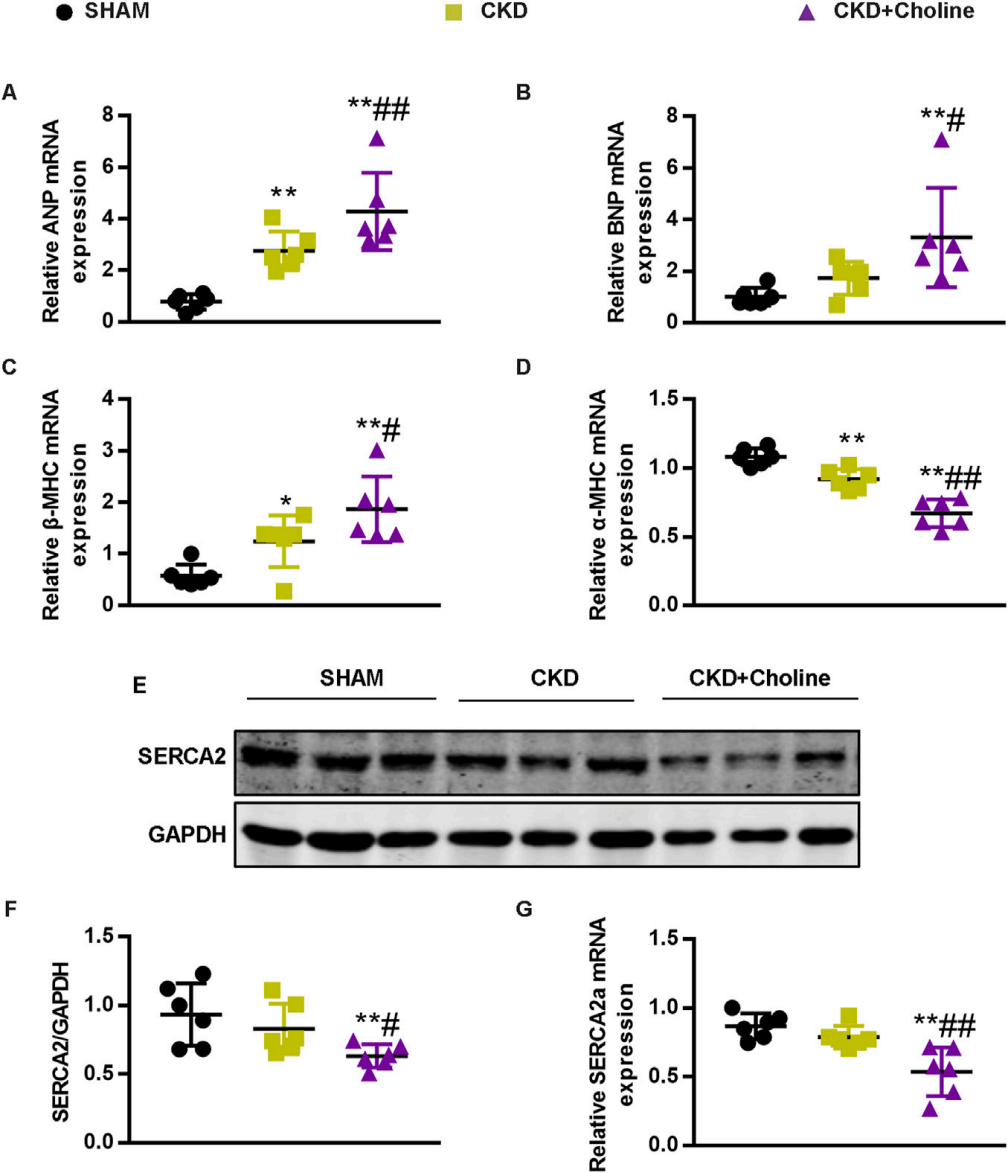
Dietary choline (1.2%) promotes changes in markers of cardiac hypertrophy and inhibits cardiac SERCA2 expression after 5/6 Nx in CD1 mice when compared with a control diet. **(A–D)** The mRNA levels of ANP, BNP, β-MHC, and α-MHC were measured by real-time PCR. **(E)** The protein levels of SERCA2 were measured by western blotting. **(F)** Graphic representation of relative abundance of SERCA2 normalized to GAPDH. **(G)** The mRNA levels of SERCA2 were measured by real-time PCR. The data are presented as the mean ± SD. ^*^ *p* < 0.05 and ^**^ *p* < 0.01 *vs.* SHAM group; ^#^ *p* < 0.05 and ^##^ *p* < 0.01 *vs.* CKD group. *n* = 6 in each group.

To further verify the effects of dietary choline in cardiac injury, we evaluated the mRNA expressions of atrial natriuretic peptide (ANP), brain natriuretic peptide (BNP), β-myosin heavy chain (β-MHC) and α-myosin heavy chain (α-MHC), which were the markers for cardiac hypertrophy (Molkentin et al., 1998). Compared with the SHAM group, ANP and β-MHC mRNA levels were significantly increased and α-MHC mRNA levels were significantly decreased in the CKD group; the increasing trend of BNP mRNA levels was also observed in the CKD group, despite no statistically difference. Meanwhile, we found that dietary choline significantly augmented the expressions of ANP, BNP, β-MHC, and significantly reduced α-MHC mRNA levels in comparison with CKD group (Figures 2A–D). Collectively, these data demonstrate a deleterious effect of dietary choline on cardiac hypertrophy.

Previous study showed that sarcoplasmic reticulum Ca2^+^-ATPase 2 (SERCA2) was down-regulated during pathological hypertrophy and heart failure, as the failing myocardium exhibits defective Ca^2+^ handling (Hasenfuss et al., 1994; Molkentin et al., 1998). To further evaluate the influence of dietary choline on cardiac function, we detected the expression of SERCA2 in mice cardiac muscle. As presented in Figures 2E–G, the protein and mRNA levels of SERCA2 were trending toward a decrease in the CKD group *versus* SHAM group, despite no statistically difference. Furthermore, 1.2% dietary choline significantly reduced the expressions of SERCA2 when compared with SHAM and CKD groups, indicating that dietary choline may affect the calcium processing of cardiomyocytes by inhibiting the expressions of SERCA2. In summary, these data support that high dietary choline aggravate LV hypertrophy and heart failure in the CKD mice.

### Medicinal charcoal tablets reduce trimethylamine-N-oxide, and improve cardiac dilatation and dysfunction in 5/6Nx mice on a high-choline diet

**FIGURE 3:**
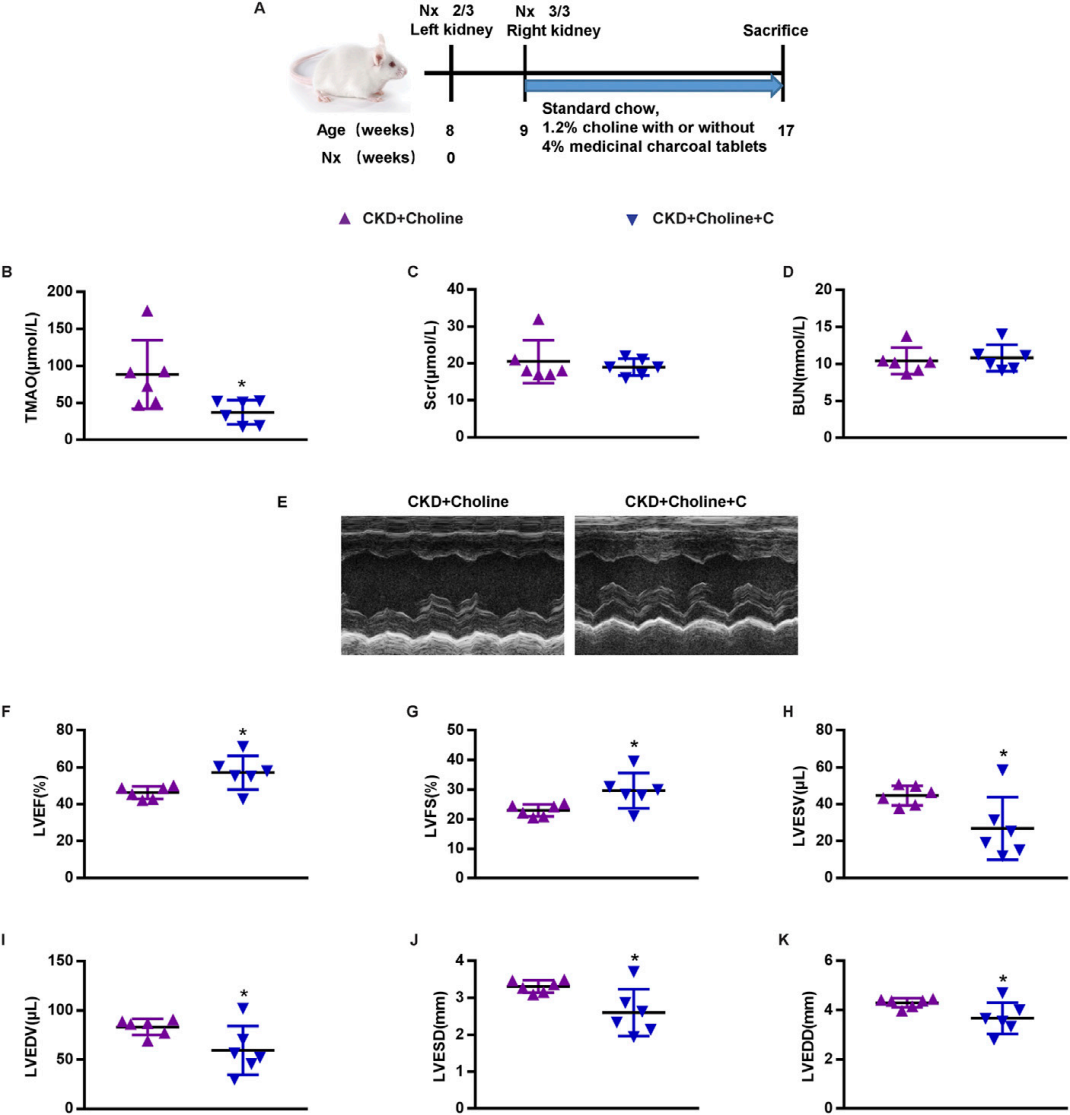
Medicinal charcoal tablets reduces TMAO, and improves cardiac dilation and dysfunction in 5/6Nx mice on a high-choline diet. **(A)** Schematic design of CKD animal model (5/6 Nx) and treatments. **(B)** The serum level of TMAO. **(C)** The serum level of Scr. **(D)** The serum level of BUN. **(E)** Representative M-mode echocardiograms for each group **(F–K)** Echocardiographic quantification of LVEF, LVFS, LVESV, LVESV, LVESD, and LVEDD. The data are presented as the mean ± SD. ^*^ *p* < 0.05 *vs.* CKD + Choline group. *n* = 6 in each group.

**FIGURE 4:**
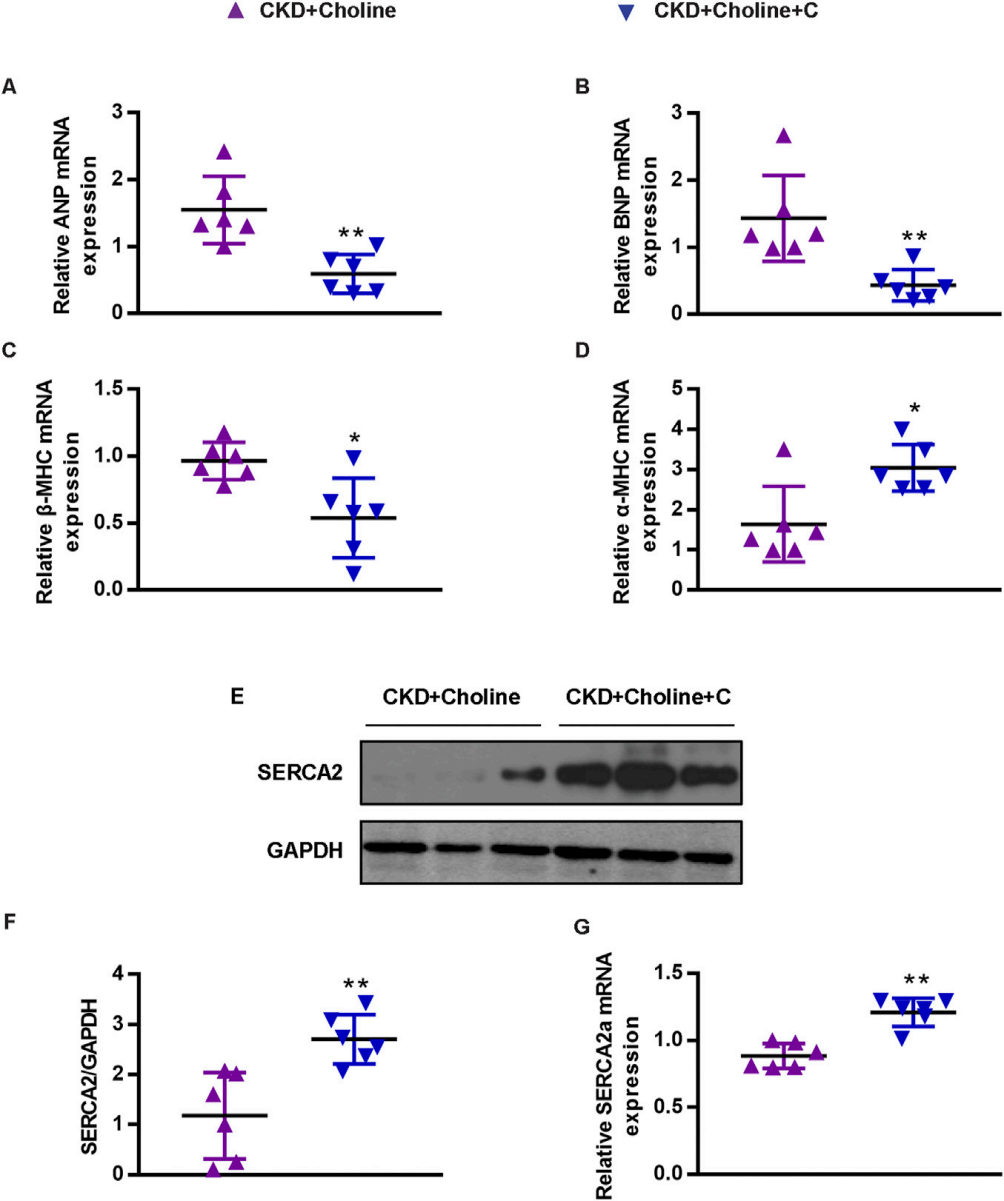
Medicinal charcoal tablets inhibit the changes of cardiac hypertrophy markers and SERCA2 expression in 5/6 Nx mice on a high-choline diet. **(A-D)** The mRNA levels of ANP, BNP, β-MHC, and α-MHC were measured by real-time PCR. **(E)** The protein levels of SERCA2 were measured by western blotting. **(F)** Graphic representation of relative abundance of SERCA2 normalized to GAPDH. **(G)** The mRNA levels of SERCA2 were measured by real-time PCR. The data are presented as the mean ± SD. ^*^ *p* < 0.05 and ^**^ *p* < 0.01 *vs.* CKD + Choline group. *n* = 6 in each group.

Medicinal charcoal tablets (MCT) are widely used in CKD patients to absorb gut uremic solutes. To determine whether reducing TMAO can improve the cardiac dysfunction in CKD mice, MCT were given orally to treat choline-supplemented mice. The complete experimental protocol for the studies was depicted in Figure 3A. As expected, we found that MCT-treated mice displayed reduced levels of TMAO when compared with CKD + Choline group, despite did not improve their renal function (Figures 3B–D). Then, cardiac structure and function in mice were assessed by echocardiography at 8 weeks post-surgery, and representative echocardiograms were shown in Figure 3E. It was revealed that MCT treatment exerted protective effects on cardiac dilatation and dysfunction, as indicated by increased LVEF and LVFS, and significant decrease in LVESD, LVEDD, LVESV, and LVEDV in mice treated with MCT compared with those without (Figures 3F–K). In addition, by MCT treatment, hypertrophic genes including ANP, BNP, and β-MHC mRNA levels were remarkably decreased, while α-MHC mRNA levels were elevated (Figures 4A–D). Furthermore, the protein and mRNA levels of the SERCA2 were remarkably increased in the MCT-treated mice when compared with the CKD + Choline group (Figures 4E–G). Taken together, MCT treatment has protective effect on heart function by improving LV structure and downregulating cardiac hypertrophic genes in choline-supplemented CKD mice.

### Dietary choline (1.2%) does not cause cardiac dilatation and dysfunction in normal CD1 mice

To test whether supplementary dietary choline could directly impair cardiac function in normal mice, we fed normal CD1 mice with a standard chow diet (0.1% choline) or high choline diet (1.2% choline). The complete experimental protocol for the studies was depicted in Supplementary Figure S2A. After 8 weeks, we observed significant increases in TMAO levels in the choline-supplemented mice when compared with control mice (12.31 ± 5.56 μmol/L *vs.* 2.50 ± 0.74 μmol/L) (Supplementary Figure S2B). However, 1.2% dietary choline did not raise the levels of Scr and BUN or cause renal histopathological impairment compared with control mice (Supplementary Figure S2C–G). Then, cardiac structure and function in mice were assessed by echocardiography, which showed no differences in LVEF, LVFS, LVESD, LVEDD, LVESV, and LVEDV between control and choline-supplemented mice (Supplementary Figure S3A–G). Furthermore, the expressions of ANP, BNP, β-MHC, α-MHC or SERCA2 (Supplementary Figure S3H–N) in the heart tissue stayed no change in the two groups. These data suggest that 1.2% dietary choline does not promote cardiac dilatation and dysfunction in normal CD1 mice.

### RNA-sequencing analysis revealed that dietary choline (1.2%) affected cardiac angiogenesis in 5/6Nx mice

**FIGURE 5:**
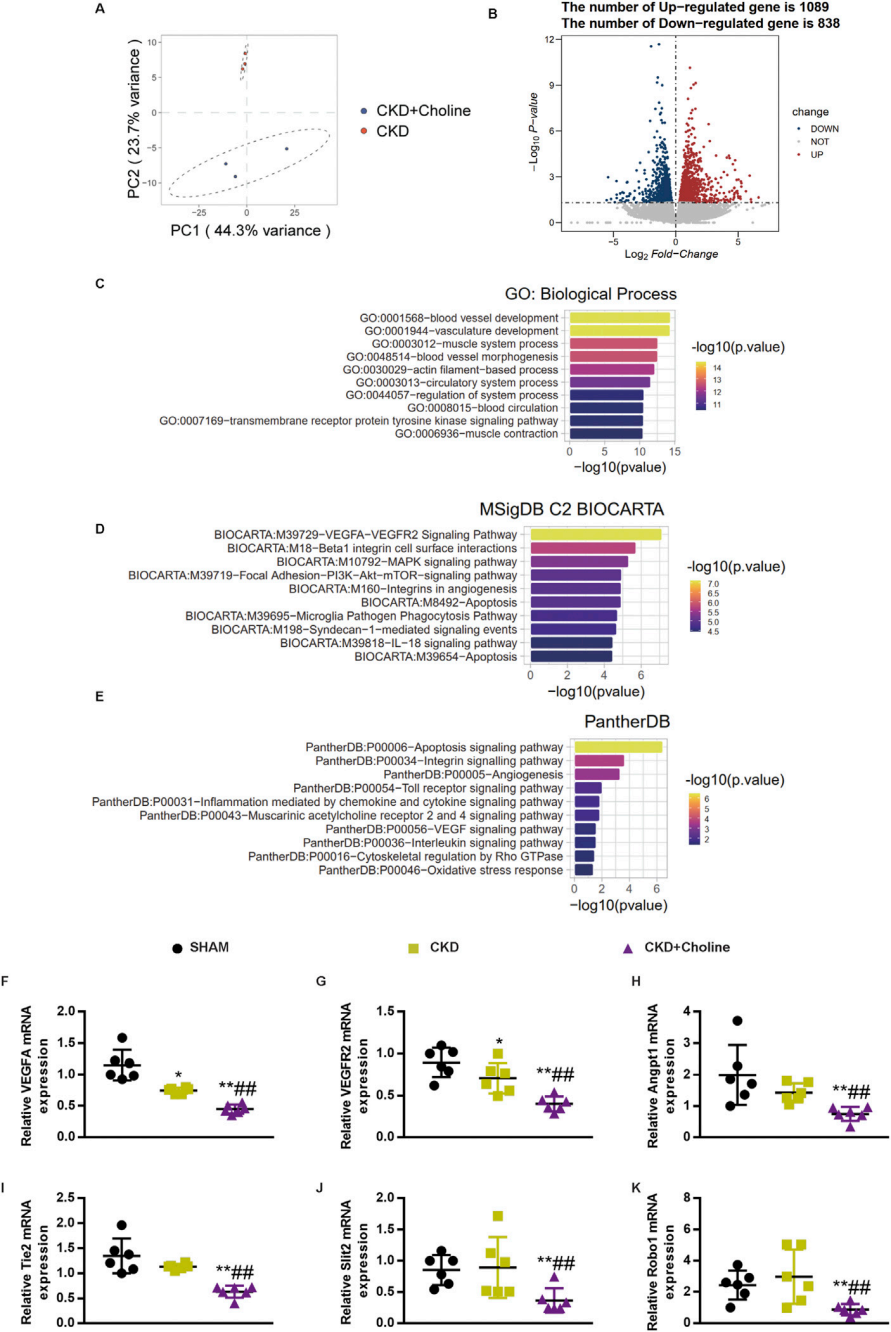
RNA-sequencing analysis revealed that dietary choline (1.2%) affected cardiac angiogenesis in 5/6Nx mice **(A)** PCA results suggested good separation between the three groups. **(B)** Volcano plot of differentially expressed genes. *X* axis: log_2_(FC); *Y* axis: -log_10_ P. Red represents upregulated genes, and blue represents downregulated genes. **(C)** GO enrichment analysis of the differentially expressed genes between CKD and CKD + Choline group **(D)** Pathway analysis the differentially expressed genes according to the MSigDB **(E)** Pathway analysis the differentially expressed genes according to the Panther program. **(F–K)** The mRNA levels of VEGF, Angpt1, Slit2, VEGFR2, Tie2 and Robo1 were measured by real-time PCR. The data are presented as the mean ± SD. ^*^ *p* < 0.05 and ^**^ *p* < 0.01 *vs.* SHAM group; ^#^ *p* < 0.05 and ^##^ *p* < 0.01 *vs.* CKD group. ^&^ *p* < 0.05 and ^&&^ *p* < 0.01 *vs.* CKD + Choline group. *n* = 6 in each group.

To explore the mechanism by which TMAO exacerbates cardiac dysfunction in CKD model, the transcriptomes of CKD and CKD + Choline groups were examined using RNA-sequencing. Principal component analysis (PCA) plots demonstrated good separation between the two data groups (Figure 5A). 1927 genes were differentially expressed (838 downregulated and 1089 upregulated) in the heart tissue of CKD + Choline mice compared with CKD mice (Figure 5B). Further GO biological process analysis showed that TMAO-regulated genes were mainly involved in blood vessel development, vasculature development, muscle system process, and blood vessel morphogenesis, *etc.* (Figure 5C). MSigDB program indicated that VEGF-VEGFR2 signaling pathway was the most significantly influenced pathway (Figure 5D). In addition, the Panther program revealed the enriched pathways of the differentially expressed genes between CKD and CKD + Choline groups, including apoptosis signaling pathway, integrin signaling pathway, angiogenesis, *etc.* (Figure 5E). We then confirmed the changes of key genes in angiogenesis in mice treated with or without dietary choline by real-time PCR. Our results suggested that 1.2% dietary choline significantly reduced the mRNA expressions of VEGF, VEGFR2, Angpt1, Tie2, Slit2, and Robo1 compared with SHAM and CKD groups (Figures 5F–K). Taken together, TMAO can aggravate cardiac dilatation and dysfunction in CKD mice mainly *via* regulating genes involved in angiogenesis.

### Dietary choline (1.2%) inhibits cardiac angiogenesis in 5/6Nx mice

**FIGURE 6:**
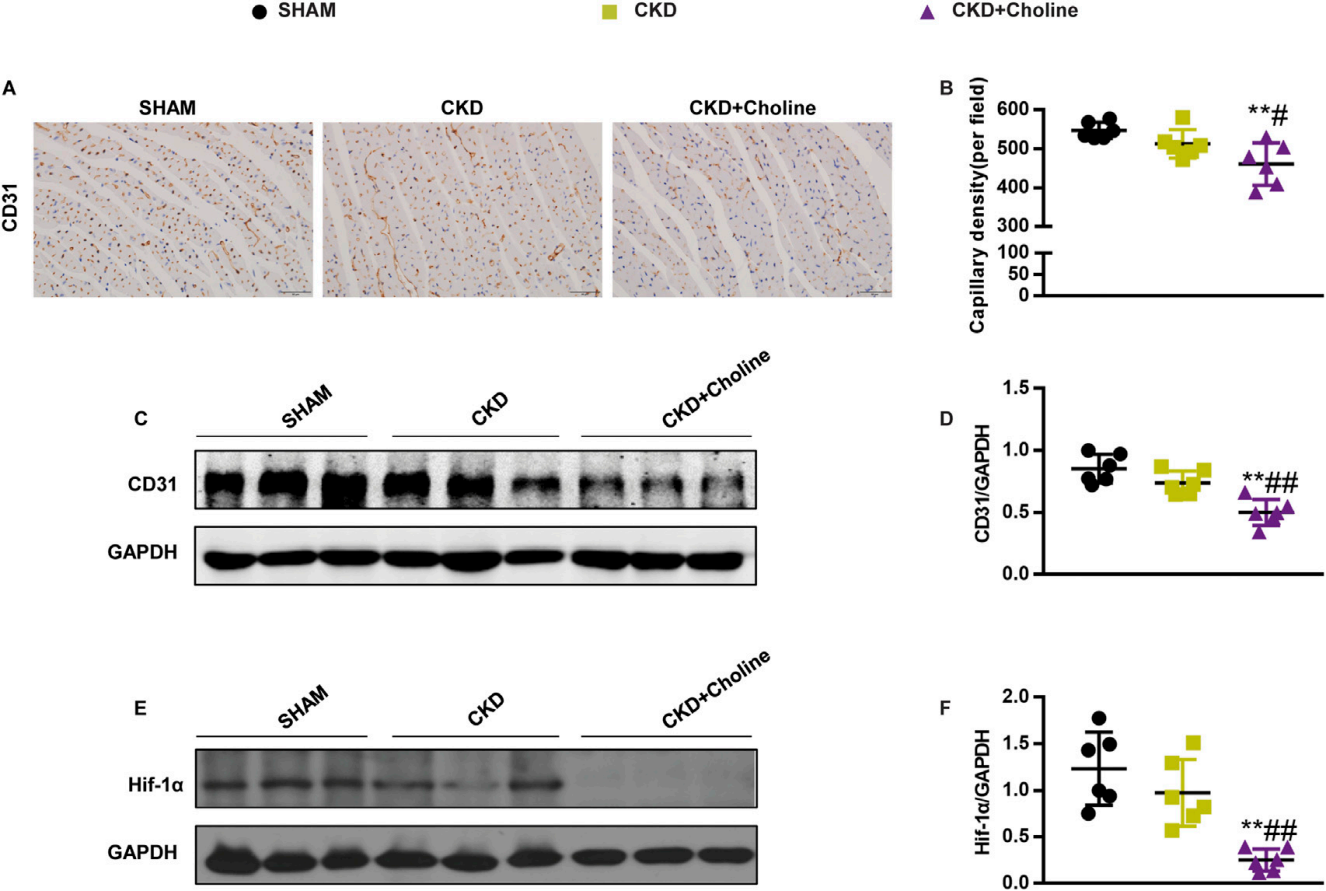
Dietary choline (1.2%) inhibits cardiac angiogenesis in 5/6Nx mice on a high-choline diet. **(A)** Representative micrographs of immunohistochemistry staining of CD31 in the heart. Scale bar = 50 μm. **(B)** Graphical representation of capillary density analysis. **(C)** The protein levels of CD31 were measured by western blotting. **(D)** Graphic representation of relative abundance of CD31 normalized to GAPDH. **(E)** The protein levels of Hif-1α were measured by western blotting. **(F)** Graphic representation of relative abundance of Hif-1α normalized to GAPDH. The data are presented as the mean ± SD. ^*^ *p* < 0.05 and ^**^ *p* < 0.01 *vs.* SHAM group; ^#^ *p* < 0.05 and ^##^ *p* < 0.01 *vs.* CKD group. ^&^ *p* < 0.05 and ^&&^ *p* < 0.01 *vs.* CKD + Choline group. *n* = 6 in each group.

As angiogenesis-related genes were found to be down-regulated in choline-supplemented CKD mice, we further examined the effect of TMAO on capillary density of heart tissues by CD31 immunohistochemical staining. As shown in Figures 6A–D, 1.2% dietary choline significantly reduced the capillary density and CD31 protein level compared with SHAM and CKD groups, although the difference between CKD and SHAM group failed to achieve statistical significance. Meanwhile, 1.2% dietary choline didn’t make differences in capillary density and CD31 levels in normal CD1 mice (Supplementary Figure S5A–D). These data support that dietary choline inhibits cardiac angiogenesis in the CKD mice.

### Dietary choline (1.2%) decreased the protein level of Hif-1α in 5/6Nx mice

Hif-1α is well-known to promote angiogenesis by regulating various angiogenesis-related genes, including VEGF (Forsythe et al., 1996), Angpt1 (Kelly et al., 2003) and Slit2 (Fang et al., 2017). Our above results showed that high choline diet could inhibit the mRNA expressions of these angiogenesis-related factors, so we speculated that high choline diet may inhibit the expression of angiogenesis-related genes by modulating the level of Hif-1α. Therefore, we examined the protein level of Hif-1α in the heart. The result revealed that the protein level of Hif-1α was trending toward a decrease in the CKD group *versus* SHAM group, despite there was no statistically difference. However, 1.2% dietary choline significantly reduced the protein level of Hif-1α when compared with SHAM and CKD groups, respectively (Figures 6E,F). These results suggest that TMAO may suppress cardiac angiogenesis by down-regulating HIF-1α.

### The Hif-1α stabilizer FG-4592 increases the expression of cardiac Hif-1α, and improves cardiac angiogenesis in 5/6Nx mice on a high-choline diet

**FIGURE 7:**
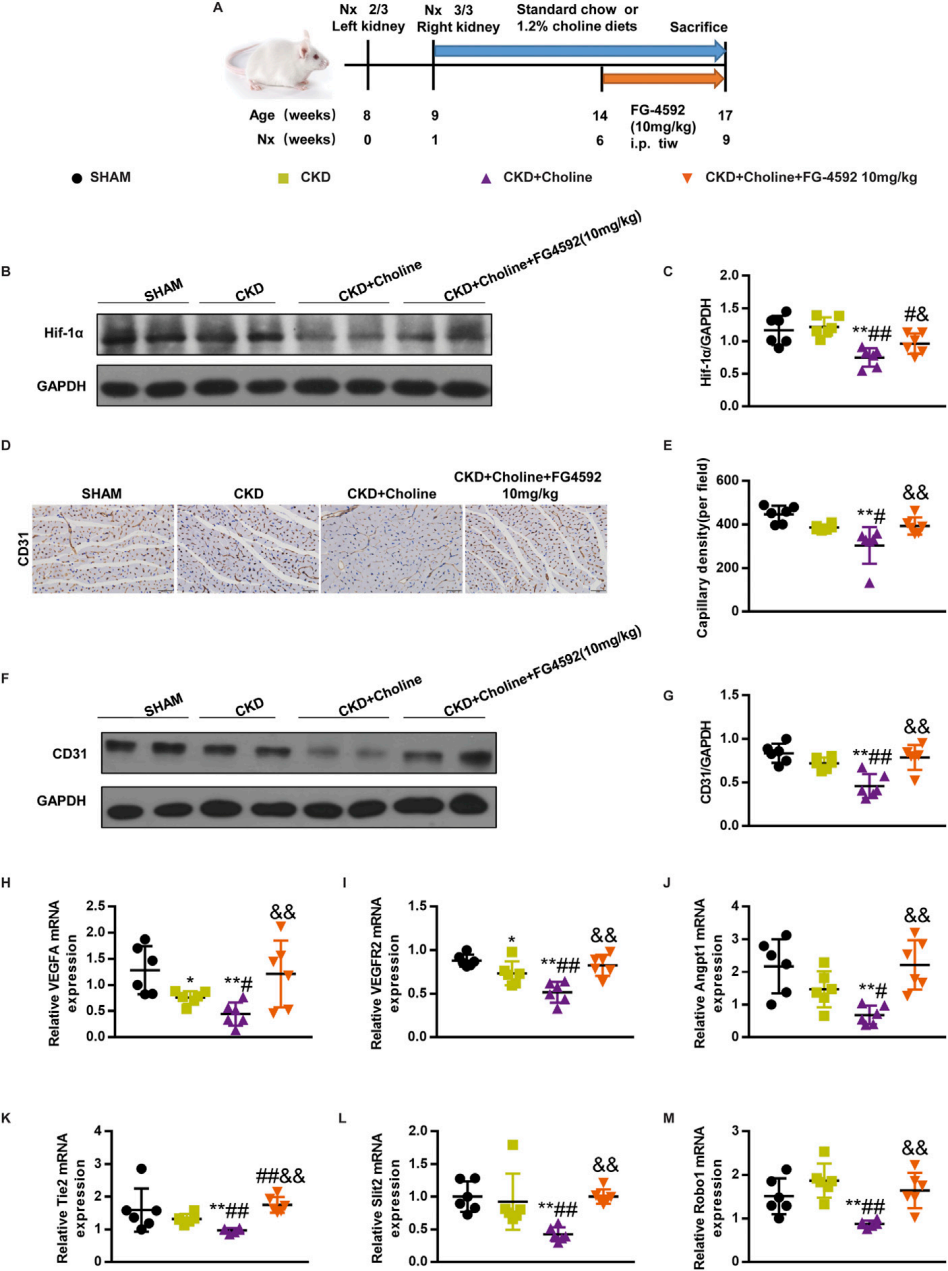
FG-4592 increases the expression of cardiac Hif-1α, and improves cardiac angiogenesis in 5/6Nx mice on a high-choline diet. **(A)** Schematic design of CKD animal model (5/6 Nx) and treatments. **(B)** The protein levels of Hif-1α were measured by western blotting. **(C)** Graphic representation of relative abundance of Hif-1α normalized to GAPDH. **(D)** Representative micrographs of immunohistochemistry staining of CD31 in the heart. Scale bar = 50 μm. **(E)** Graphical representation of capillary density analysis. **(F)** The protein levels of CD31 were measured by western blotting. **(G)** Graphic representation of relative abundance of CD31 normalized to GAPDH. **(H–M)** The mRNA levels of VEGF, Angpt1, Slit2, VEGFR2, Tie2 and Robo1 were measured by real-time PCR. The data are presented as the mean ± SD. ^*^ *p* < 0.05 and ^**^ *p* < 0.01 *vs.* SHAM group; ^#^ *p* < 0.05 and ^##^ *p* < 0.01 *vs.* CKD group. ^&^ *p* < 0.05 and ^&&^ *p* < 0.01 *vs.* CKD + Choline group *n* = 6 in each group.

To evaluate whether elevating Hif-1α improves cardiac angiogenesis in 5/6Nx mice fed by high-choline diet, we treated them with FG-4592, a stabilizer of Hif-1α, which has been used to treat renal anemia in CKD patients (Voit and Sankaran, 2020; Hu et al., 2021). From 5 weeks after surgery, CKD + Choline group was sub-divided into two groups, receiving intraperitoneal injection of FG-4592 (10 mg/kg) or PBS every day. The complete experimental protocol for the studies was depicted in Figure 7A. Western blot confirmed that FG-4592 treatment prominently upregulated the protein level of Hif-1α when compared with CKD + Choline group (Figures 7B,C). Then we detected indicators of anemia including hemoglobin (Hb), red blood cell (RBC) count, and hematocrit (Hct). Consistent with previous study, FG-4592 ameliorated anemia caused by renal dysfunction in the CKD mice (Supplementary Figure S5A–C). Next, we explored the effect of FG-4592 on capillary density by CD31 immunohistochemical staining. The reduction of CD31-positive endothelial capillaries in CKD + Choline group was reversed by FG-4592 treatment (Figures 7D,E). In addition, FG-4592 treatment restored angiogenesis related genes including VEGF, VEGFR2, Angpt1, Tie2, Slit2, and Robo expression in the heart (Figures 7H–M). Collectively, these data demonstrate that FG-4592 elevates cardiac Hif-1α protein level and improves cardiac angiogenesis in 5/6Nx mice fed with high-choline diet.

### FG-4592 does not reduce trimethylamine-N-oxide, but improves cardiac dilatation and dysfunction in 5/6Nx mice on a high-choline diet

**FIGURE 8:**
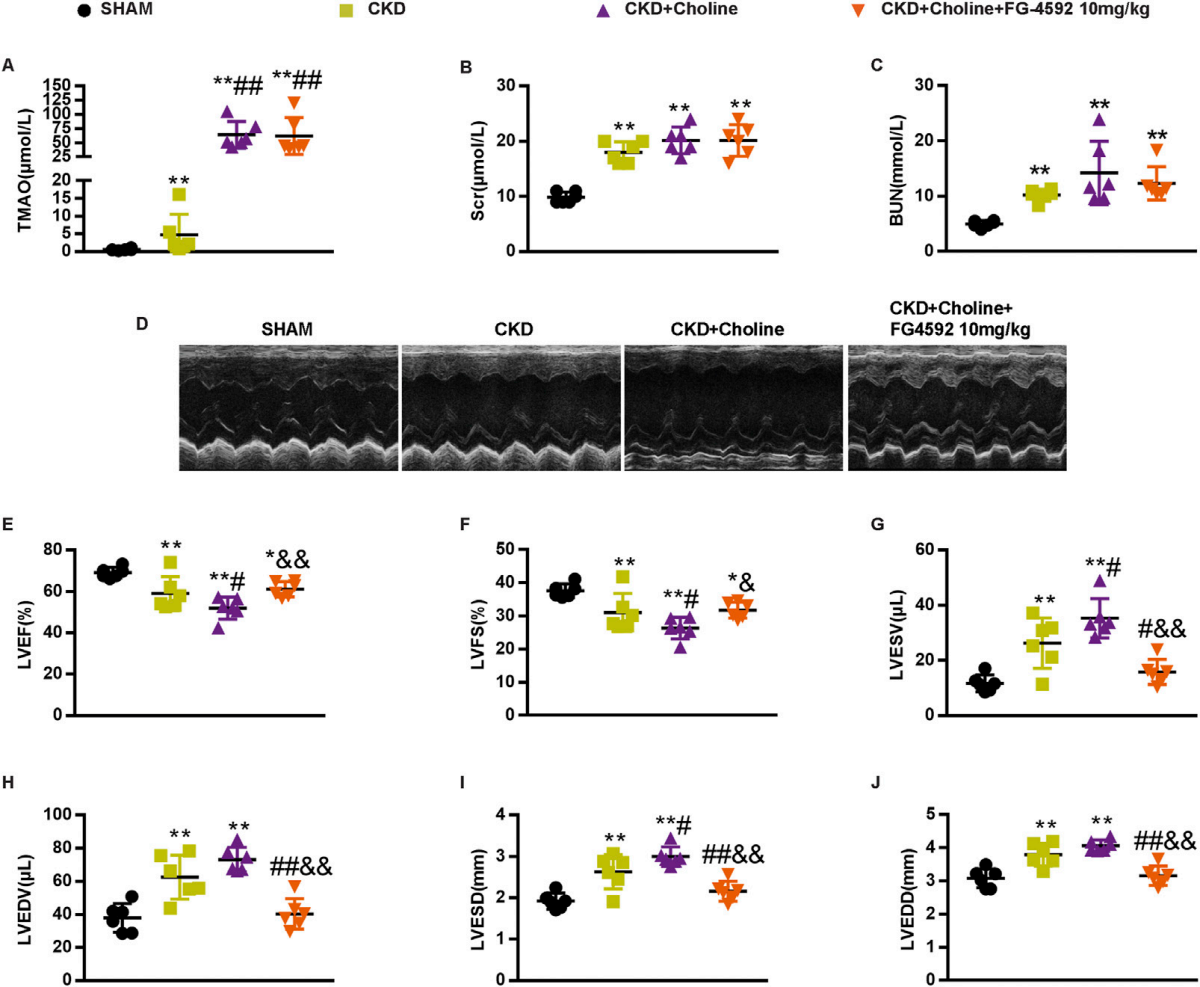
FG-4592 does not alter the level of TMAO, but improves cardiac dilation and dysfunction in 5/6 Nx mice on a high-choline diet. **(A)** The serum level of TMAO. **(B)** The serum level of Scr. **(C)** The serum level of BUN. **(D)** Representative M-mode echocardiograms for each group. **(E–J)** Echocardiographic quantification of LVEF, LVFS, LVESV, LVESV, LVESD, and LVEDD. The data are presented as the mean ± SD. ^*^ *p* < 0.05 and ^**^ *p* < 0.01 *vs.* SHAM group; ^#^ *p* < 0.05 and ^##^ *p* < 0.01 *vs.* CKD group. ^&^ *p* < 0.05 and ^&&^ *p* < 0.01 *vs.* CKD + Choline group *n* = 6 in each group.

**FIGURE 9:**
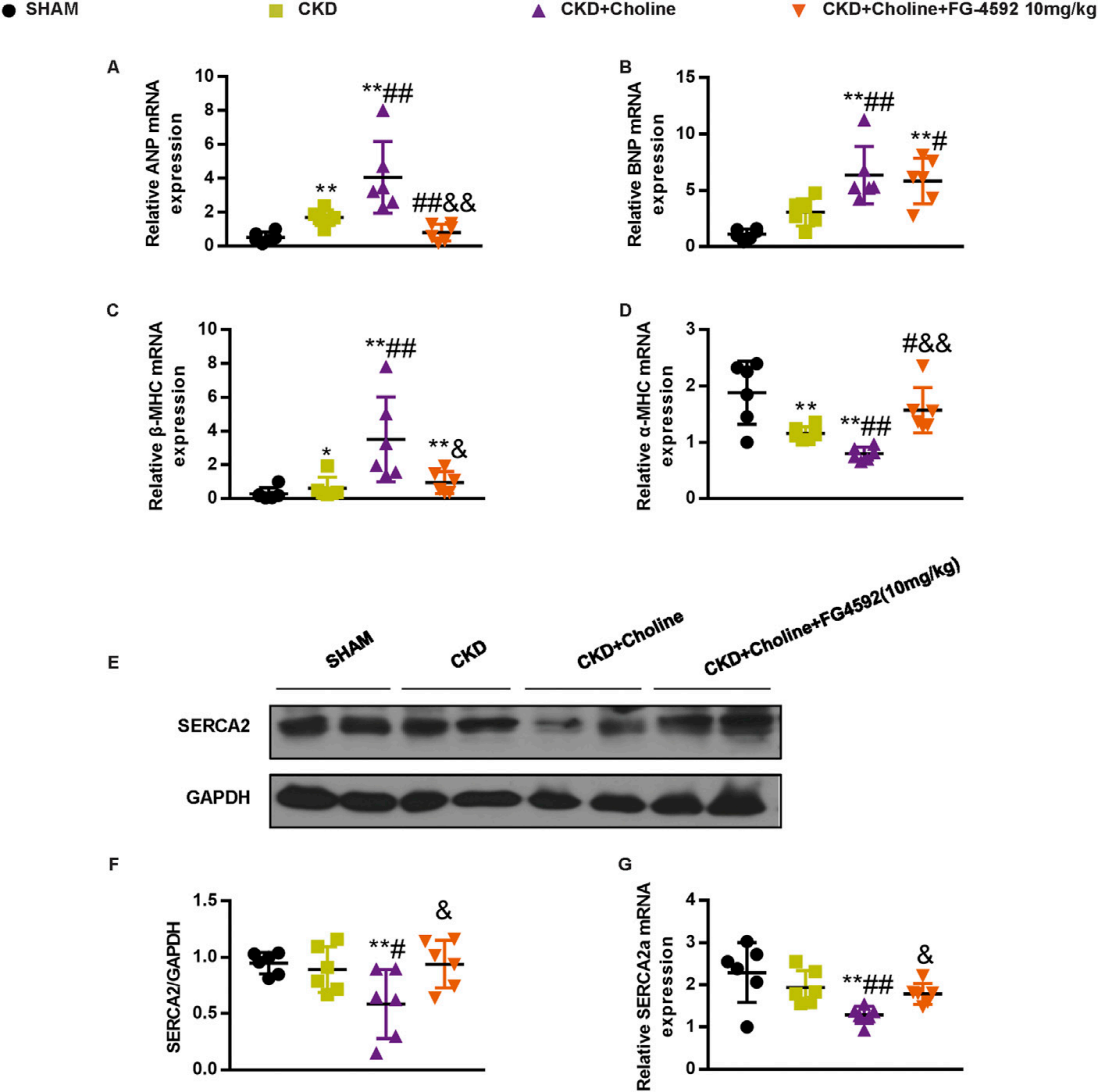
FG-4592 inhibits the changes of cardiac hypertrophy markers and SERCA2 expression in 5/6 Nx mice on a high-choline diet. **(A–D)** The mRNA levels of ANP, BNP, β-MHC, and α-MHC were measured by real-time PCR. **(E)** The protein levels of SERCA2 were measured by western blotting. **(F)** Graphic representation of relative abundance of SERCA2 normalized to GAPDH. **(G)** The mRNA levels of SERCA2 were measured by real-time PCR. The data are presented as the mean ± SD. ^*^ *p* < 0.05 and ^**^ *p* < 0.01 *vs.* SHAM group; ^#^ *p* < 0.05 and ^##^ *p* < 0.01 *vs.* CKD group; ^&^ *p* < 0.05 and ^&&^ *p* < 0.01 *vs.* CKD + Choline group. *n* = 6 in each group.

As cardiac angiogenesis is closely related to heart function, we evaluate the effect of FG-4592 treatment on cardiac function in the CKD mice by echocardiography (Figure 8D). The results revealed that FG-4592 rescued the reduction of LVEF and LVFS as well as the elevation of LVESD, LVEDD, LVESV, and LVEDV in CKD + Choline group (Figures 8E–J), indicating that FG-4592 attenuates cardiac dilatation and dysfunction caused by CKD and choline-supplement. Then, real-time PCR analysis showed that ANP and β-MHC mRNA levels were remarkably decreased and α-MHC mRNA levels were increased in the FG-4592-treated mice when compared with the CKD + Choline group (Figures 9A,C,D). Although a trend toward decrease in BNP mRNA level was observed in the FG-4592-treated mice, this difference failed to achieve statistical significance (Figure 9B). The levels of the SERCA2 were also prominently increased in the FG-4592-treated mice compared with CKD + Choline group (Figures 9E–G). In addition, our data showed that FG-4592 treatment improved cardiac function without affecting TMAO levels or improving renal function (Figures 8A–C). In summary, these findings indicate that Hif-1α stabilizer FG-4592 attenuates dietary choline-induced cardiac dilatation and dysfunction in the setting of CKD, *via* improving cardiac angiogenesis.

## Discussion

Accumulating studies have shown strong associations between TMAO and adverse cardiovascular risks in non-CKD (Tang et al., 2013; Tang et al., 2015b; Troseid et al., 2015; Nie et al., 2018) and CKD patients (Kim et al., 2016; Shafi et al., 2017; Fu et al., 2021). Several *in vivo* studies also revealed that either choline- or TMAO-supplemented diets led to adverse cardiac dysfunction in a mouse model of myocardial infarction (Yang et al., 2019) or transverse aortic constriction (TAC) (Organ et al., 2016). Unlike cardiac infarction, the mechanisms of CKD-related cardiac dysfunction are more complex, involving in anemia, hypertension, hemodynamic overload, mineral-bone disease, as well as damage of numerous uremic toxins (Garikapati et al., 2021). However, few studies to date have examined the role of TMAO in the progression of CKD-induced cardiac dysfunction. In this study, we fed CKD mice with high-choline diet to further increase their serum concentration of TMAO (89.78 ± 31.57 μmol/L) to mimic the situation of CKD patients (Shafi et al., 2017). We found that dietary choline aggravated cardiac dysfunction in CKD mice, which could be markedly improved by reducing TMAO levels *via* MCT treatment. This result provides evidence that TMAO is responsible for CKD-induced cardiac dysfunction. However, we do not exclude the possibility that various mechanisms such as hypertension, volume overload, oxidative stress, secondary hyperparathyroidism and other gut microbiota toxins such as IS and PCS are also involved in the pathogenesis of CKD-related heart diseases. It is noteworthy that MCT does not only decrease TMAO but also clear other enteric toxins such as IS and PCS (data not shown). Even so, our data support that MCT treatment exerts cardioprotective effects at least partially through decreasing TMAO level.

It is well-known that impairment of kidney can accelerate the progression of cardiac dysfunction (Shamseddin and Parfrey, 2009). In the current study, dietary choline did not worsen renal function in CKD mice. Additionally, MCT ameliorated cardiac dysfunction without affecting renal function, indicating that the effect of TMAO on aggravating cardiac dysfunction is not dependent on the deterioration of renal injury in CKD mice. However, previous study reported that dietary choline or TMAO caused renal tubulointerstitial fibrosis in normal C57BL/6J mice (Tang et al., 2015a). This discrepancy may be explained by differences in the initial state of the kidneys when receiving dietary choline. Indeed, although dietary choline for 8 weeks did not exacerbate kidney damage in normal CD1 mice, when we extended dietary choline administration to 12 weeks, modest tubular damage and increased BUN level were found (data not shown).

Several studies have reported the mechanisms by which TMAO promotes CVD including promoting foam cell formation (Wang et al., 2011), vascular inflammation (Seldin et al., 2016), and platelet activation (Zhu et al., 2016). In this study, we conducted RNA-seq to explore the mechanism by which TMAO promotes cardiac injury in CKD mice. GO enrichment analysis revealed that TMAO-regulated genes are mainly involved in blood vessel development, vasculature development and blood vessel morphogenesis. MSigDB program further indicated that VEGF-VEGFR2 signaling pathway was the most significantly influenced pathway. Consistently, reduced cardiac vascular density and decreased expression of angiogenesis-related genes, including VEGF, VEGFR2, Angpt1, Tie2, Slit2, and Robo1, were observed in cardiac tissue in CKD mice fed with a high-choline diet. In support of our finding, the inhibitory effects of TMAO on endothelial cell proliferation, migration, and tube formation have been observed by *in vitro* studies (Ma et al., 2017; Ke et al., 2018). In addition, recent studies have demonstrated that TMAO impairs perfusion recovery and reduces capillary density after hindlimb ischemia (Chen et al., 2020; Liu et al., 2021). Collectively, these data support a role of TMAO in interfering with angiogenesis.

Extensive evidence have confirmed the protective effect of HIF-1α on the heart, including promoting capillaries restoration, improving Ca^2+^ handling and inhibiting fibroblasts proliferation (Janbandhu et al., 2022). HIF-1α plays a critical role in angiogenesis by activating transcription of genes encoding angiogenic growth factors including VEGF (Forsythe et al., 1996), Angpt1 (Kelly et al., 2003) and Slit2 (Fang et al., 2017). Reduced Hif-1α protein is one of the critical mechanisms that underlies exacerbated myocardial hypoxia and accelerated myocardial damage and dysfunction (Sano et al., 2007). Sano et al. (2007) found that deletion of Hif-1α in cardiomyocytes resulted in significant decrease in myocardial vascular density and remarkable cardiac dysfunction in TAC mice, which improved by elevating Hif-1α protein levels. Similarly, we observed that dietary choline lowered cardiac Hif-1α protein levels in CKD mice, and Hif-1α stabilizer FG-4592 improved cardiac angiogenesis. These findings suggest that dietary choline inhibits cardiac angiogenesis in CKD mice by decreasing cardiac Hif-1α protein levels.

Previous study showed that Hif-1α^+/−^ mice developed more severe heart failure after TAC compared with wild type mice, due to a decrease in SR Ca^2+^ content of cardiomyocytes (Silter et al., 2010). Indeed, we also detected the downregulation of SERCA2 expression in cardiac tissue of mice fed with high choline diet. Besides, our RNA-seq analyses revealed that high-choline diet upregulated the apoptosis signaling pathway in cardiac tissues. Indeed, we observed that dietary choline elevated the protein expression of p53 in the myocardium of CKD mice (data not shown). Previous study showed that p53 participated in hypoxia-induced cardiomyocyte apoptosis (Long et al., 1997). Since RNA-seq analysis showed that blood vessel development, vasculature development and blood vessel morphogenesis were the most significantly influenced pathway by high choline diet, we hypothesized that TMAO-induced vascular rarefaction initiates ischemic and hypoxic cardiomyocyte dysfunction and apoptosis. It is noteworthy that although cardiac Hif-1α protein levels in the CKD mice was not significantly reduced compared with SHAM group, cardiac dysfunction was observed in CKD mice. Indeed, besides insufficient angiogenesis, other mechanisms such as hypertension, volume overload, activation of the RAS system, and insulin resistance are also involved in CKD-induced cardiac injury (Wang and Shapiro, 2019).

So far, treatment of UC mainly focus on lipid-lowering, anti-hypertension and anemia correction, which only modestly improved cardiovascular outcomes (Garikapati et al., 2021). Notably, our results demonstrated that FG-4592, a novel approved clinical treatment of renal anemia, improved angiogenesis and cardiac dysfunction in CKD mice. These data suggest FG-4592 is beneficial to cardiac function in CKD patients and this benefit does not solely come from correcting anemia. Our data provided positive evidence for the clinical application of FG-4592 in patients with UC. Currently, the clinical trial that evaluating the potential effect of FG-4592 on cardio-renal syndromes in CKD patients is under recruitment (NCT05053893).

In summary, the present findings extend associations between TMAO and cardiovascular risk in CKD patients, by demonstrating a remarkable adverse effect of dietary choline on CKD-induced cardiac dysfunction. Besides, targeting TMAO might be a potential new therapeutic approach for UC. Furthermore, FG-4592 might be a promising drug for improving UC besides its role in treating CKD anemia.

## Data Availability

The datasets presented in this study can be found in online repositories. The names of the repository/repositories and accession number(s) can be found below: https://bigd.big.ac.cn/gsa/, CRA007956.

## Supplementary material

Supplementary Figure S1 Dietary choline (1.2%) does not alter renal injury and fibrosis after 5/6 Nx in CD1 mice. **(A)** Representative micrographs of H&E (top row, scale bar = 100 μm) and Masson Trichrome staining (bottom row, scale bar = 50 μm) show the morphology of kidney. **(B)** Tubular injury score. **(C)** The fibrotic region (blue stain) quantified as the percentage of the total area. The data are presented as the mean ± SD. **p* <0.05 and ***p* < 0.01 *vs.* SHAM group; #*p* < 0.05 and ##*p* < 0.01 *vs.* CKD group. *n* = 6 in each group.

Supplementary Figure S2 Dietary choline (1.2%) increases the serum level of TMAO, but not alters kidney morphology in CD1 mice. **(A)** Schematic design of treatments. **(B)** The serum level of TMAO. **(C)** The serum level of Scr. **(D)** The serum level of BUN. **(E)** Representative micrographs of H&E (top row, scale bar = 100 μm) and Masson Trichrome staining (bottom row, scale bar = 50 μm) show the morphology of kidney. **(F)** Tubular injury score. **(G)** The fibrotic region (blue stain) quantified as the percentage of the total area. The data are presented as the mean ± SD. **p* <0.05 and ***p* < 0.01 *vs.* CON group. *n* = 6 in each group.

Supplementary Figure S3 Dietary choline (1.2%) does not affect cardiac function and morphology in CD1 mice. **(A)** Representative M-mode echocardiograms for each group. **(B–G)** Echocardiographic quantification of LVEF, LVFS, LVESV, LVESV, LVESD, and LVEDD. **(H)** Photomicrographs of left ventricular tissue sections stained with hematoxylin and eosin (H&E). **(I–L)** The mRNA levels of β-MHC, ANP, BNP, and SERCA2a were measured by real-time PCR. **(M)** The protein levels of SERCA2 were measured by western blotting. **(N)** Graphic representation of relative abundance of SERCA2 normalized to GAPDH. The data are presented as the mean ± SD. **p* <0.05 and ***p* < 0.01 *vs.* CON group. *n* = 6 in each group.

Supplementary Figure S4 Dietary choline (1.2%) does not affect cardiac capillary density in CD1 mice. **(A)** Representative micrographs of immunohistochemistry staining of CD31 in the heart. Scale bar = 50 μm. **(B)** Graphical representation of capillary density analysis. **(C)** The protein levels of CD31 were measured by western blotting. **(D)** Graphic representation of relative abundance of CD31 normalized to GAPDH. The data are presented as the mean ± SD. *n* = 6 in each group.

Supplementary Figure S5 FG-4592 elevates Hb, RBC and Hct in 5/6Nx mice on a high-choline diet. **(A–C)** Hb **(A)**, RBC **(B)** and Hct (C) were detected in blood collected from mice at the end point of experiment. The data are presented as the mean ± SD. **p* <0.05 and ***p* < 0.01 *vs.* SHAM group; #*p* < 0.05 and ##*p* < 0.01 *vs.* CKD group. ^&^ *p* < 0.05 and ^&&^ *p* < 0.01 *vs.* CKD+Choline group. *n* = 6 in each group. Hb, Hemoglobin; RBC, Red blood cell; Hct, Hematocrit.

## References

[B1] Amann K. Breitbach M. Ritz E. Mall G. (1998). Myocyte/capillary mismatch in the heart of uremic patients. J. Am. Soc. Nephrol. 9, 1018–1022. 10.1681/ASN.V961018 9621284

[B2] Amann K. Wiest G. Zimmer G. Gretz N. Ritz E. Mall G. (1992). Reduced capillary density in the myocardium of uremic rats--a stereological study. Kidney Int. 42, 1079–1085. 10.1038/ki.1992.390 1453595

[B3] Bi X. Yang K. Zhang B. Zhao J. (2020). The protective role of klotho in CKD-associated cardiovascular disease. Kidney Dis. 6, 395–406. 10.1159/000509369 PMC7706511 33313060

[B4] Breier G. Damert A. Plate K. H. Risau W. (1997). Angiogenesis in embryos and ischemic diseases. Thromb. Haemost. 78, 678–683. 10.1055/s-0038-1657611 9198238

[B5] Chen L. Jin Y. Wang N. Yuan M. Lin T. Lu W. (2020). Trimethylamine N-oxide impairs perfusion recovery after hindlimb ischemia. Biochem. Biophys. Res. Commun. 530, 95–99. 10.1016/j.bbrc.2020.06.093 32828321

[B57] Collins A. J. Foley R. N. Gilbertson D. T. Chen S. C. (2015). United States Renal Data System public health surveillance of chronic kidney disease and end-stage renal disease. Kidney Int. Suppl. (2011) 5 (1), 2–7. 10.1038/kisup.2015.2 26097778 PMC4455192

[B6] Dou L. Bertrand E. Cerini C. Faure V. Sampol J. Vanholder R. (2004). The uremic solutes p-cresol and indoxyl sulfate inhibit endothelial proliferation and wound repair. Kidney Int. 65, 442–451. 10.1111/j.1523-1755.2004.00399.x 14717914

[B7] Fang M. Du H. Han B. Xia G. Shi X. Zhang F. (2017). Hypoxia-inducible microRNA-218 inhibits trophoblast invasion by targeting LASP1: Implications for preeclampsia development. Int. J. Biochem. Cell Biol. 87, 95–103. 10.1016/j.biocel.2017.04.005 28412444

[B8] Fang Q. Zheng B. Liu N. Liu J. Liu W. Huang X. (2021). Trimethylamine N-oxide exacerbates renal inflammation and fibrosis in rats with diabetic kidney disease. Front. Physiol. 12, 682482. 10.3389/fphys.2021.682482 34220546 PMC8243655

[B9] Forsythe J. A. Jiang B. H. Iyer N. V. Agani F. Leung S. W. Koos R. D. (1996). Activation of vascular endothelial growth factor gene transcription by hypoxia-inducible factor 1. Mol. Cell. Biol. 16, 4604–4613. 10.1128/MCB.16.9.4604 8756616 PMC231459

[B10] Fu D. Shen J. Li W. Wang Y. Zhong Z. Ye H. (2021). Elevated serum trimethylamine N-oxide levels are associated with mortality in male patients on peritoneal dialysis. Blood Purif. 50, 837–847. 10.1159/000512962 33596582

[B11] Garikapati K. Goh D. Khanna S. Echampati K. (2021). Uraemic cardiomyopathy: A review of current literature. Clin. Med. Insights. Cardiol. 15, 1179546821998347. 10.1177/1179546821998347 33707979 PMC7907931

[B12] GBD Chronic Kidney Disease Collaboration (2020). Global, regional, and national burden of chronic kidney disease, 1990-2017: a systematic analysis for the global burden of disease study 2017. Lancet 395, 709–733. 10.1016/S0140-6736(20)30045-3 32061315 PMC7049905

[B13] Green D. Roberts P. R. New D. I. Kalra P. A. (2011). Sudden cardiac death in hemodialysis patients: an in-depth review. Am. J. Kidney Dis. 57, 921–929. 10.1053/j.ajkd.2011.02.376 21496983

[B14] Gschwend S. Buikema H. Navis G. Henning R. H. De Zeeuw D. Van Dokkum R. P. (2002). Endothelial dilatory function predicts individual susceptibility to renal damage in the 5/6 nephrectomized rat. J. Am. Soc. Nephrol. 13, 2909–2915. 10.1097/01.asn.0000036865.22253.d4 12444209

[B15] Hartiala J. Bennett B. J. Tang W. H. Wang Z. Stewart A. F. Roberts R. (2014). Comparative genome-wide association studies in mice and humans for trimethylamine N-oxide, a proatherogenic metabolite of choline and L-carnitine. Arterioscler. Thromb. Vasc. Biol. 34, 1307–1313. 10.1161/ATVBAHA.114.303252 24675659 PMC4035110

[B16] Hasenfuss G. Reinecke H. Studer R. Meyer M. Pieske B. Holtz J. (1994). Relation between myocardial function and expression of sarcoplasmic reticulum Ca(2+)-ATPase in failing and nonfailing human myocardium. Circ. Res. 75, 434–442. 10.1161/01.res.75.3.434 8062417

[B17] Hsu B. G. Wang C. H. Lin Y. L. Lai Y. H. Tsai J. P. (2022). Serum trimethylamine N-oxide level is associated with peripheral arterial stiffness in advanced non-dialysis chronic kidney disease patients. Toxins (Basel) 14, 526. 10.3390/toxins14080526 36006188 PMC9414425

[B18] Hu X. Xie J. Chen N. (2021). Hypoxia-Inducible factor-proline hydroxylase inhibitor in the treatment of renal anemia. Kidney Dis. (Basel). 7, 1–9. 10.1159/000510587 33614728 PMC7879335

[B19] Hung S. C. Kuo K. L. Huang H. L. Lin C. C. Tsai T. H. Wang C. H. (2016). Indoxyl sulfate suppresses endothelial progenitor cell-mediated neovascularization. Kidney Int. 89, 574–585. 10.1016/j.kint.2015.11.020 26880454

[B20] Hung S. C. Kuo K. L. Wu C. C. Tarng D. C. (2017). Indoxyl sulfate: A novel cardiovascular risk factor in chronic kidney disease. J. Am. Heart Assoc. 6, e005022. 10.1161/JAHA.116.005022 28174171 PMC5523780

[B21] Hung S. C. Lai Y. S. Kuo K. L. Tarng D. C. (2015). Volume overload and adverse outcomes in chronic kidney disease: clinical observational and animal studies. J. Am. Heart Assoc. 4, e001918. 10.1161/JAHA.115.001918 25944876 PMC4599419

[B22] Janbandhu V. Tallapragada V. Patrick R. Li Y. Abeygunawardena D. Humphreys D. T. (2022). Hif-1a suppresses ROS-induced proliferation of cardiac fibroblasts following myocardial infarction. Cell Stem Cell 29, 281–297. 10.1016/j.stem.2021.10.009 34762860 PMC9021927

[B23] Ke Y. Li D. Zhao M. Liu C. Liu J. Zeng A. (2018). Gut flora-dependent metabolite Trimethylamine-N-oxide accelerates endothelial cell senescence and vascular aging through oxidative stress. Free Radic. Biol. Med. 116, 88–100. 10.1016/j.freeradbiomed.2018.01.007 29325896

[B24] Kelly B. D. Hackett S. F. Hirota K. Oshima Y. Cai Z. Berg-Dixon S. (2003). Cell type-specific regulation of angiogenic growth factor gene expression and induction of angiogenesis in nonischemic tissue by a constitutively active form of hypoxia-inducible factor 1. Circ. Res. 93, 1074–1081. 10.1161/01.RES.0000102937.50486.1B 14576200

[B25] Kennedy D. J. Elkareh J. Shidyak A. Shapiro A. P. Smaili S. Mutgi K. (2008). Partial nephrectomy as a model for uremic cardiomyopathy in the mouse. Am. J. Physiol. Ren. Physiol. 294, F450–F454. 10.1152/ajprenal.00472.2007 PMC2742580 18032546

[B26] Kim R. B. Morse B. L. Djurdjev O. Tang M. Muirhead N. Barrett B. (2016). Advanced chronic kidney disease populations have elevated trimethylamine N-oxide levels associated with increased cardiovascular events. Kidney Int. 89, 1144–1152. 10.1016/j.kint.2016.01.014 27083288

[B27] Lai Y. Tang H. Zhang X. Zhou Z. Zhou M. Hu Z. (2022). Trimethylamine-N-Oxide aggravates kidney injury via activation of p38/MAPK signaling and upregulation of HuR. Kidney Blood Press. Res. 47, 61–71. 10.1159/000519603 34788763

[B28] Leelahavanichkul A. Yan Q. Hu X. Eisner C. Huang Y. Chen R. (2010). Angiotensin II overcomes strain-dependent resistance of rapid CKD progression in a new remnant kidney mouse model. Kidney Int. 78, 1136–1153. 10.1038/ki.2010.287 20736988 PMC3113489

[B29] Li T. Gua C. Wu B. Chen Y. (2018). Increased circulating trimethylamine N-oxide contributes to endothelial dysfunction in a rat model of chronic kidney disease. Biochem. Biophys. Res. Commun. 495, 2071–2077. 10.1016/j.bbrc.2017.12.069 29247650

[B30] Liabeuf S. Barreto D. V. Barreto F. C. Meert N. Glorieux G. Schepers E. (2010). Free p-cresylsulphate is a predictor of mortality in patients at different stages of chronic kidney disease. Nephrol. Dial. Transpl. 25, 1183–1191. 10.1093/ndt/gfp592 19914995

[B31] Liu X. Shao Y. Tu J. Sun J. Dong B. Wang Z. (2021). TMAO-activated hepatocyte-derived exosomes impair angiogenesis via repressing CXCR4. Front. Cell Dev. Biol. 9, 804049. 10.3389/fcell.2021.804049 35174166 PMC8841965

[B32] London G. M. (2003). Cardiovascular disease in chronic renal failure: pathophysiologic aspects. Semin. Dial. 16, 85–94. 10.1046/j.1525-139x.2003.16023.x 12641870

[B33] Long X. Boluyt M. O. Hipolito M. L. Lundberg M. S. Zheng J. S. O'Neill L. (1997). p53 and the hypoxia-induced apoptosis of cultured neonatal rat cardiac myocytes. J. Clin. Invest. 99, 2635–2643. 10.1172/JCI119452 9169493 PMC508109

[B34] Lu D. Wang K. Wang S. Zhang B. Liu Q. Zhang Q. (2017). Beneficial effects of renal denervation on cardiac angiogenesis in rats with prolonged pressure overload. Acta Physiol. 220, 47–57. 10.1111/apha.12793 27575955

[B35] Ma G. Pan B. Chen Y. Guo C. Zhao M. Zheng L. (2017). Trimethylamine N-oxide in atherogenesis: impairing endothelial self-repair capacity and enhancing monocyte adhesion. Biosci. Rep. 37, BSR20160244. 10.1042/BSR20160244 28153917 PMC5333780

[B36] Molkentin J. D. Lu J. R. Antos C. L. Markham B. Richardson J. Robbins J. (1998). A calcineurin-dependent transcriptional pathway for cardiac hypertrophy. Cell 93, 215–228. 10.1016/s0092-8674(00)81573-1 9568714 PMC4459646

[B37] Nakano T. Katsuki S. Chen M. Decano J. L. Halu A. Lee L. H. (2019). Uremic toxin indoxyl sulfate promotes proinflammatory macrophage activation via the interplay of OATP2B1 and dll4-notch signaling. Circulation 139, 78–96. 10.1161/CIRCULATIONAHA.118.034588 30586693 PMC6311723

[B38] Nie J. Xie L. Zhao B. X. Li Y. Qiu B. Zhu F. (2018). Serum trimethylamine N-oxide concentration is positively associated with first stroke in hypertensive patients. Stroke 49, 2021–2028. 10.1161/STROKEAHA.118.021997 30354996

[B39] Organ C. L. Otsuka H. Bhushan S. Wang Z. Bradley J. Trivedi R. (2016). Choline diet and its gut microbe-derived metabolite, trimethylamine N-oxide, exacerbate pressure overload-induced heart failure. Circ. Heart Fail. 9, e002314. 10.1161/CIRCHEARTFAILURE.115.002314 26699388 PMC4943035

[B40] Roberts A. B. Gu X. Buffa J. A. Hurd A. G. Wang Z. Zhu W. (2018). Development of a gut microbe-targeted nonlethal therapeutic to inhibit thrombosis potential. Nat. Med. 24, 1407–1417. 10.1038/s41591-018-0128-1 30082863 PMC6129214

[B41] Sano M. Minamino T. Toko H. Miyauchi H. Orimo M. Qin Y. (2007). p53-induced inhibition of Hif-1 causes cardiac dysfunction during pressure overload. Nature 446, 444–448. 10.1038/nature05602 17334357

[B42] Seldin M. M. Meng Y. Qi H. Zhu W. Wang Z. Hazen S. L. (2016). Trimethylamine N-oxide promotes vascular inflammation through signaling of mitogen-activated protein kinase and nuclear factor-κb. J. Am. Heart Assoc. 5, e002767. 10.1161/JAHA.115.002767 26903003 PMC4802459

[B43] Semple D. Smith K. Bhandari S. Seymour A. M. (2011). Uremic cardiomyopathy and insulin resistance: a critical role for akt? J. Am. Soc. Nephrol. 22, 207–215. 10.1681/ASN.2009090900 20634295

[B44] Shafi T. Powe N. R. Meyer T. W. Hwang S. Hai X. Melamed M. L. (2017). Trimethylamine N-oxide and cardiovascular events in hemodialysis patients. J. Am. Soc. Nephrol. 28, 321–331. 10.1681/ASN.2016030374 27436853 PMC5198291

[B45] Shamseddin M. K. Parfrey P. S. (2009). Mechanisms of the cardiorenal syndromes. Nat. Rev. Nephrol. 5, 641–649. 10.1038/nrneph.2009.156 19786991

[B46] Shuai W. Wen J. Li X. Wang D. Li Y. Xiang J. (2020). High-choline diet exacerbates cardiac dysfunction, fibrosis, and inflammation in a mouse model of heart failure with preserved ejection fraction. J. Card. Fail. 26, 694–702. 10.1016/j.cardfail.2020.04.017 32417378

[B47] Silter M. Kogler H. Zieseniss A. Wilting J. Schafer K. Toischer K. (2010). Impaired Ca(2+)-handling in HIF-1alpha(+/-) mice as a consequence of pressure overload. Pflugers Arch. 459, 569–577. 10.1007/s00424-009-0748-x 19898976 PMC2827795

[B48] Stubbs J. R. House J. A. Ocque A. J. Zhang S. Johnson C. Kimber C. (2016). Serum trimethylamine-N-oxide is elevated in CKD and correlates with coronary atherosclerosis burden. J. Am. Soc. Nephrol. 27, 305–313. 10.1681/ASN.2014111063 26229137 PMC4696571

[B49] Subramaniam S. Fletcher C. (2018). Trimethylamine N-oxide: breathe new life. Br. J. Pharmacol. 175, 1344–1353. 10.1111/bph.13959 28745401 PMC5866995

[B50] Suzuki T. Heaney L. M. Jones D. J. Ng L. L. (2017). Trimethylamine N-oxide and risk stratification after acute myocardial infarction. Clin. Chem. 63, 420–428. 10.1373/clinchem.2016.264853 28062632

[B51] Tabibiazar R. Rockson S. G. (2001). Angiogenesis and the ischaemic heart. Eur. Heart J. 22, 903–918. 10.1053/euhj.2000.2372 11428814

[B52] Tang W. H. Wang Z. Kennedy D. J. Wu Y. Buffa J. A. Agatisa-Boyle B. (2015a). Gut microbiota-dependent trimethylamine N-oxide (TMAO) pathway contributes to both development of renal insufficiency and mortality risk in chronic kidney disease. Circ. Res. 116, 448–455. 10.1161/CIRCRESAHA.116.305360 25599331 PMC4312512

[B53] Tang W. H. Wang Z. Levison B. S. Koeth R. A. Britt E. B. Fu X. (2013). Intestinal microbial metabolism of phosphatidylcholine and cardiovascular risk. N. Engl. J. Med. 368, 1575–1584. 10.1056/NEJMoa1109400 23614584 PMC3701945

[B54] Tang W. H. Wang Z. Shrestha K. Borowski A. G. Wu Y. Troughton R. W. (2015b). Intestinal microbiota-dependent phosphatidylcholine metabolites, diastolic dysfunction, and adverse clinical outcomes in chronic systolic heart failure. J. Card. Fail. 21, 91–96. 10.1016/j.cardfail.2014.11.006 25459686 PMC4312712

[B55] Tonelli M. Wiebe N. Culleton B. House A. Rabbat C. Fok M. (2006). Chronic kidney disease and mortality risk: a systematic review. J. Am. Soc. Nephrol. 17, 2034–2047. 10.1681/ASN.2005101085 16738019

[B56] Troseid M. Ueland T. Hov J. R. Svardal A. Gregersen I. Dahl C. P. (2015). Microbiota-dependent metabolite trimethylamine-N-oxide is associated with disease severity and survival of patients with chronic heart failure. J. Intern. Med. 277, 717–726. 10.1111/joim.12328 25382824

[B58] Voit R. A. Sankaran V. G. (2020). Stabilizing HIF to ameliorate anemia. Cell 180, 6. 10.1016/j.cell.2019.12.010 31951520

[B59] Wang X. Liu J. Drummond C. A. Shapiro J. I. (2017). Sodium potassium adenosine triphosphatase (Na/K-ATPase) as a therapeutic target for uremic cardiomyopathy. Expert Opin. Ther. Targets 21, 531–541. 10.1080/14728222.2017.1311864 28338377 PMC5590225

[B60] Wang X. Shapiro J. I. (2019). Evolving concepts in the pathogenesis of uraemic cardiomyopathy. Nat. Rev. Nephrol. 15, 159–175. 10.1038/s41581-018-0101-8 30664681

[B61] Wang Z. Klipfell E. Bennett B. J. Koeth R. Levison B. S. Dugar B. (2011). Gut flora metabolism of phosphatidylcholine promotes cardiovascular disease. Nature 472, 57–63. 10.1038/nature09922 21475195 PMC3086762

[B62] Wang Z. Levison B. S. Hazen J. E. Donahue L. Li X. M. Hazen S. L. (2014). Measurement of trimethylamine-N-oxide by stable isotope dilution liquid chromatography tandem mass spectrometry. Anal. Biochem. 455, 35–40. 10.1016/j.ab.2014.03.016 24704102 PMC4167037

[B63] Wang Z. Roberts A. B. Buffa J. A. Levison B. S. Zhu W. Org E. (2015). Non-lethal inhibition of gut microbial trimethylamine production for the treatment of atherosclerosis. Cell 163, 1585–1595. 10.1016/j.cell.2015.11.055 26687352 PMC4871610

[B64] Yang K. Du C. Wang X. Li F. Xu Y. Wang S. (2017). Indoxyl sulfate induces platelet hyperactivity and contributes to chronic kidney disease-associated thrombosis in mice. Blood 129, 2667–2679. 10.1182/blood-2016-10-744060 28264799

[B65] Yang W. Zhang S. Zhu J. Jiang H. Jia D. Ou T. (2019). Gut microbe-derived metabolite trimethylamine N-oxide accelerates fibroblast-myofibroblast differentiation and induces cardiac fibrosis. J. Mol. Cell. Cardiol. 134, 119–130. 10.1016/j.yjmcc.2019.07.004 31299216

[B66] Zhang Z. Y. Hu C. F. Wang M. X. Lin J. Li J. M. Wang R. Z. (2018). Research on mechanism of PCS in damaging vascular endothelial cells and promoting formation of atherosclerosis via TLR4/TREM-1. Eur. Rev. Med. Pharmacol. Sci. 22, 7533–7542. 10.26355/eurrev_201811_16295 30468503

[B67] Zhu W. Gregory J. C. Org E. Buffa J. A. Gupta N. Wang Z. (2016). Gut microbial metabolite TMAO enhances platelet hyperreactivity and thrombosis risk. Cell 165, 111–124. 10.1016/j.cell.2016.02.011 26972052 PMC4862743

[B68] Zoccali C. (2010). Left ventricular systolic dysfunction: a sudden killer in end-stage renal disease patients. Hypertension 56, 187–188. 10.1161/HYPERTENSIONAHA.110.151829 20606109

