# Supplementary figures and images for: Dietary choline, via gut microbe- generated trimethylamine-N- oxide, aggravates chronic kidney disease-induced cardiac dysfunction by inhibiting hypoxia-induced factor 1α
Source: Front Physiol. 2022 Nov 3;13:996166. doi: 10.3389/fphys.2022.996166 (PMC9669413; doi:10.3389/fphys.2022.996166)

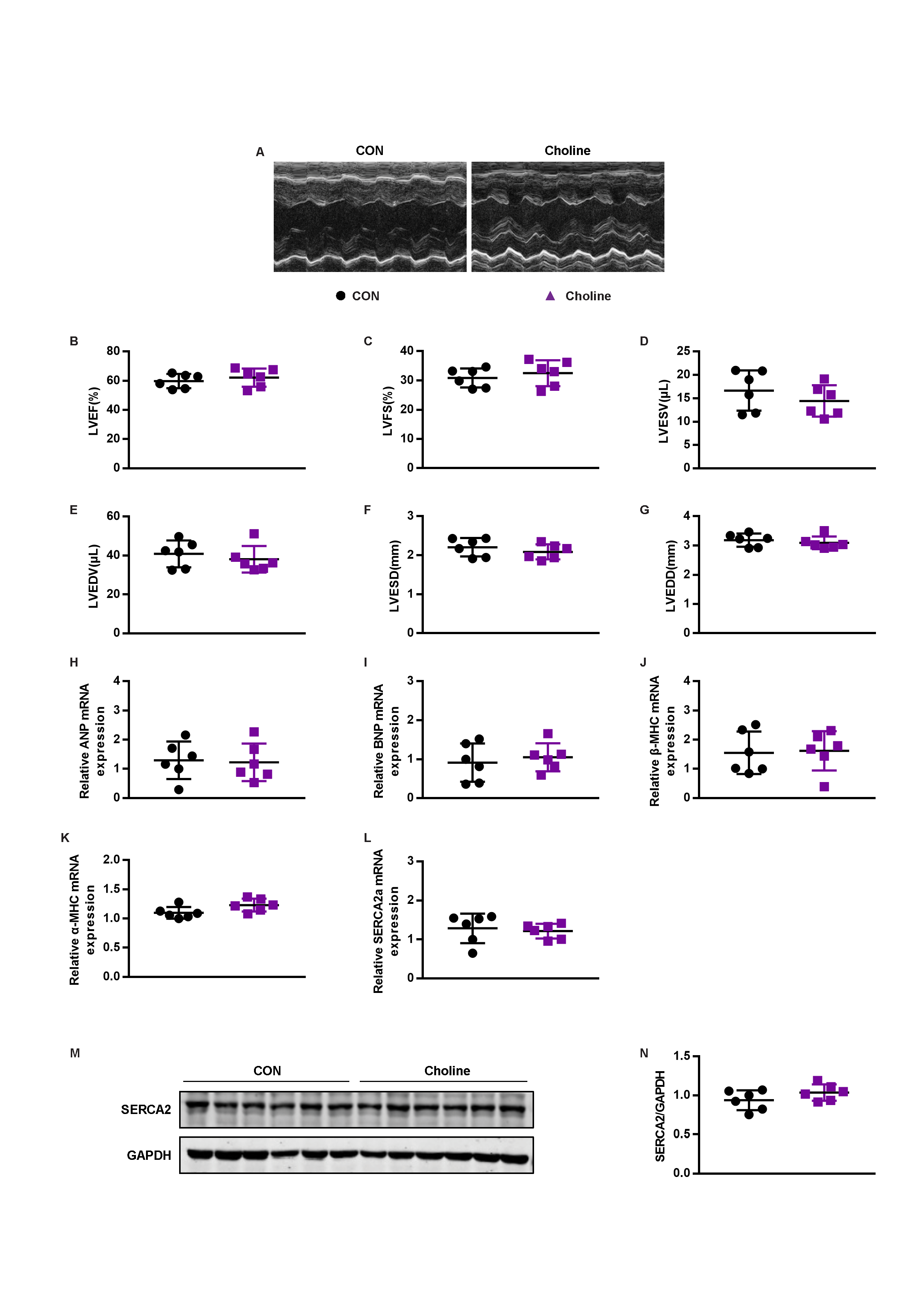

Supplement: Supplementary file 1 [file Image3.TIF]

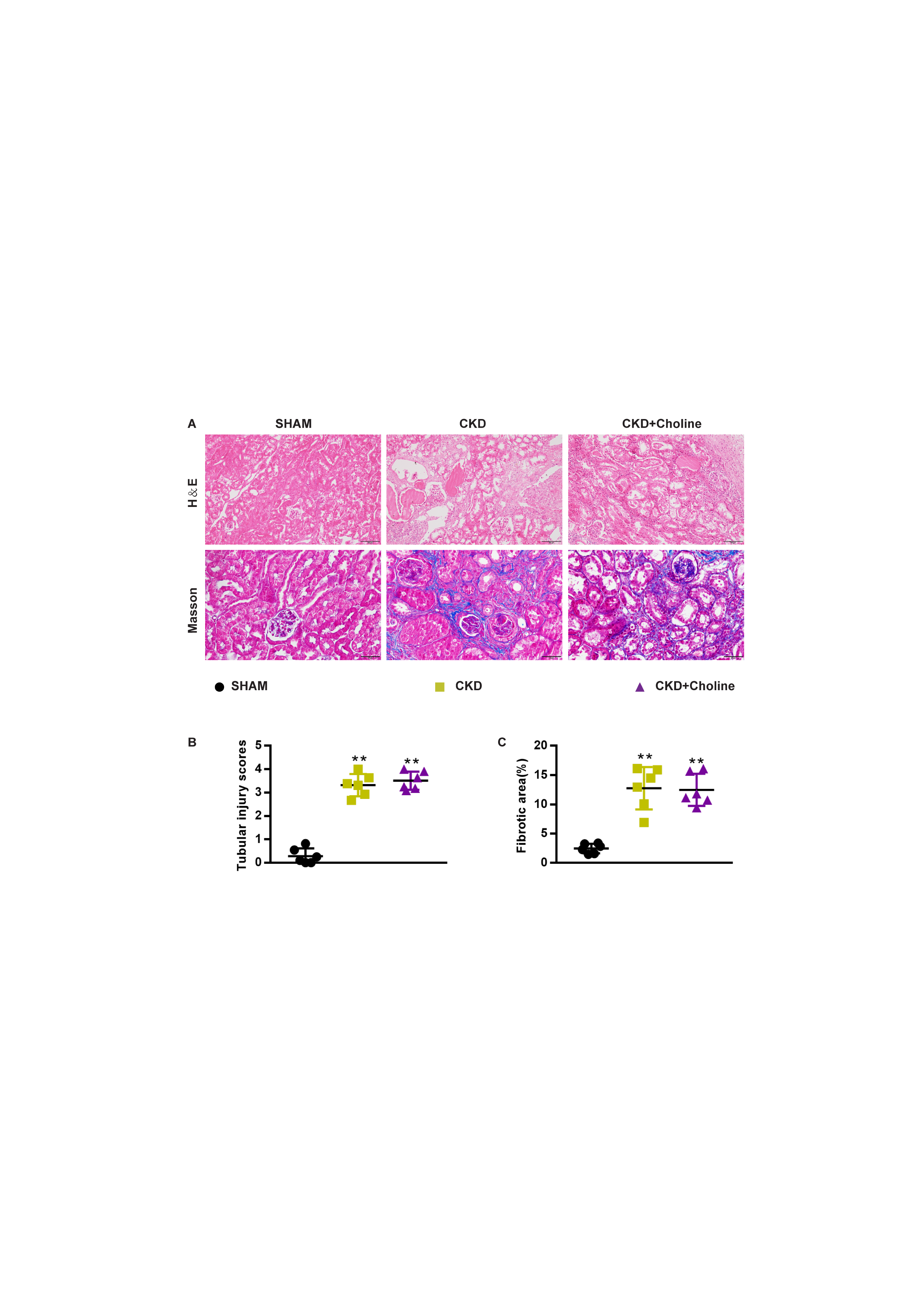

Supplement: Supplementary file 4 [file Image1.TIF]

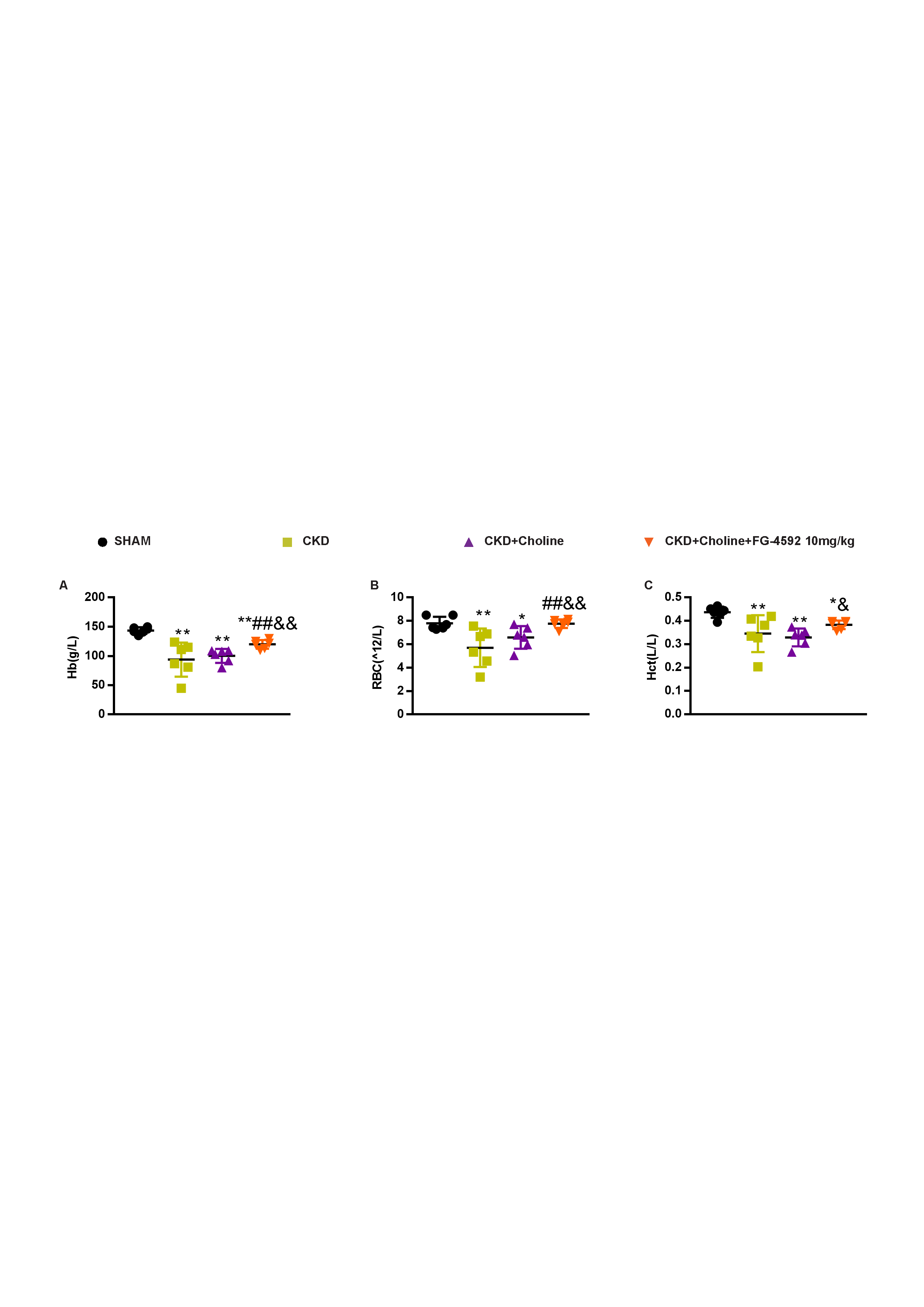

Supplement: Supplementary file 5 [file Image5.TIF]
